# Catalytic p*K*_a_ Attenuation in a Hydrolytic Metalloenzyme by Genetic Code Expansion

**DOI:** 10.1021/acs.biochem.5c00768

**Published:** 2026-02-18

**Authors:** Benjamin P. Manser, Alexandria Deliz Liang

**Affiliations:** Department of Chemistry, 27217 University of Zurich, Winterthurerstrasse 190, 8057 Zurich, Switzerland

## Abstract

Hydrolytic metalloenzymes employ Lewis-acidic metal cofactors to activate water molecules, generating nucleophilic hydroxide species that facilitate catalysis. Their catalytic efficiency across a wide pH range is often governed by the protonation state of the metal-bound water, reflected in p*K*_a_ values typically between 6.8 and 9. Modulating this parameter is key to expanding enzymatic activity for improved activity at neutral to acidic pH. Herein, we apply genetic code expansion to mutate the primary metal-coordination sphere of a model metallohydrolase: the dizinc phosphotriesterase from *Pseudomonas diminuta*. Substitution of the most catalytically indispensable coordinating histidine residue (H55) to *N* ^π^-methyl-l-histidine (πMH) resulted in substantial enzyme yields, efficient metal coordination for either Zn^2+^ or Co^2+^, and up to 5-fold improved tolerance to acidic conditions. Detailed mechanistic analysis revealed a systematic decrease in catalytic p*K*_a_ and attenuation of several catalytic rate constants. These results add to the growing body of evidence demonstrating the power of ncAA-based engineering for refined tuning of enzyme properties.

## Graphical abstract

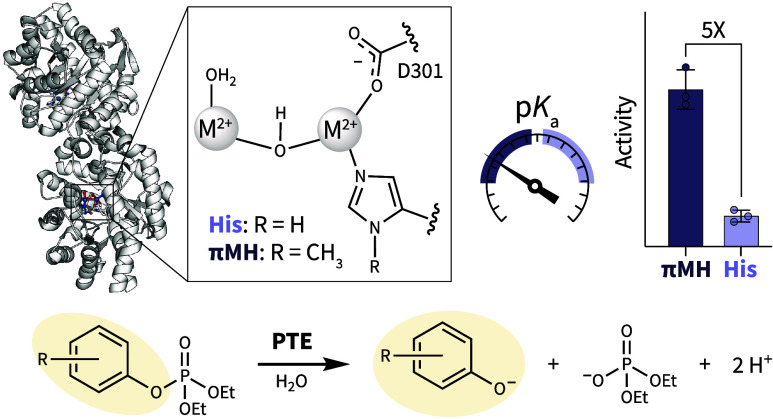

## Introduction

Metallohydrolases are metal-dependent enzymes that catalyze hydrolytic reactions through activation of a water molecule, increasing the nucleophilic character of the oxygen atom by facilitating deprotonation or forming a strong hydrogen bonding interaction.^1^ Often, hydrolytic enzymes exhibit their optimal activity at neutral to alkaline pH, exhibiting catalytic p*K*_a_s typically within pH 6.8 to 9.^2–4^ These catalytic p*K*_a_s are proposed to reflect the protonation state of the metal-bound water.^2–4^ At lower pH values, the deprotonation of the catalytic water molecule is impaired, resulting in loss of activity. Thus, decreasing the p*K*_a_ of this catalytic water molecule could improve catalytic function at neutral and low pH. The p*K*_a_ of the metal-bound water molecule is affected by the metal identity, the primary coordination sphere ligands, and the secondary sphere interactions through hydrogen bonding or hydrophobic interactions.^5–7^ Thus, the ability to probe each of these features is valuable for understanding and engineering these catalysts for improved function.

Bacterial phosphotriesterases (PTEs) represent a well-studied subgroup of hydrolytic metalloenzymes. PTEs were first identified in *Pseudomonas diminuta* (dPTE) and *Flavobacterium sp*. collected from insecticide-contaminated soil and were found to catalyze the hydrolytic degradation of organophosphates such as the insecticide paraoxon (Figure 1A) as well as several nerve agents such as sarin and VX. Thus, the enzyme family has attracted attention as potential bioremediation and decontamination candidates.^8–10^ dPTE has a binuclear metal-site with a histidine-rich binding motif (Figure 1B).^11^ Each metal is coordinated by two histidine residues, and the two metals are bridged by a carboxylated lysine and a hydroxide ion. The α-metal is additionally coordinated by an aspartic acid residue; and the β-metal is additionally coordinated by a water molecule, which is reportedly displaced by the substrate during catalysis. The proposed mechanism involves ligand exchange at the β-metal, releasing water and enabling substrate binding, which orients the phosphorus center near the bridging hydroxide species.^12–14^ The hydroxide performs a nucleophilic attack, facilitated by the aspartate coordinated to the α-metal and resulting in the release of the leaving group (Figure 1C).^14,15^

**Figure 1:**
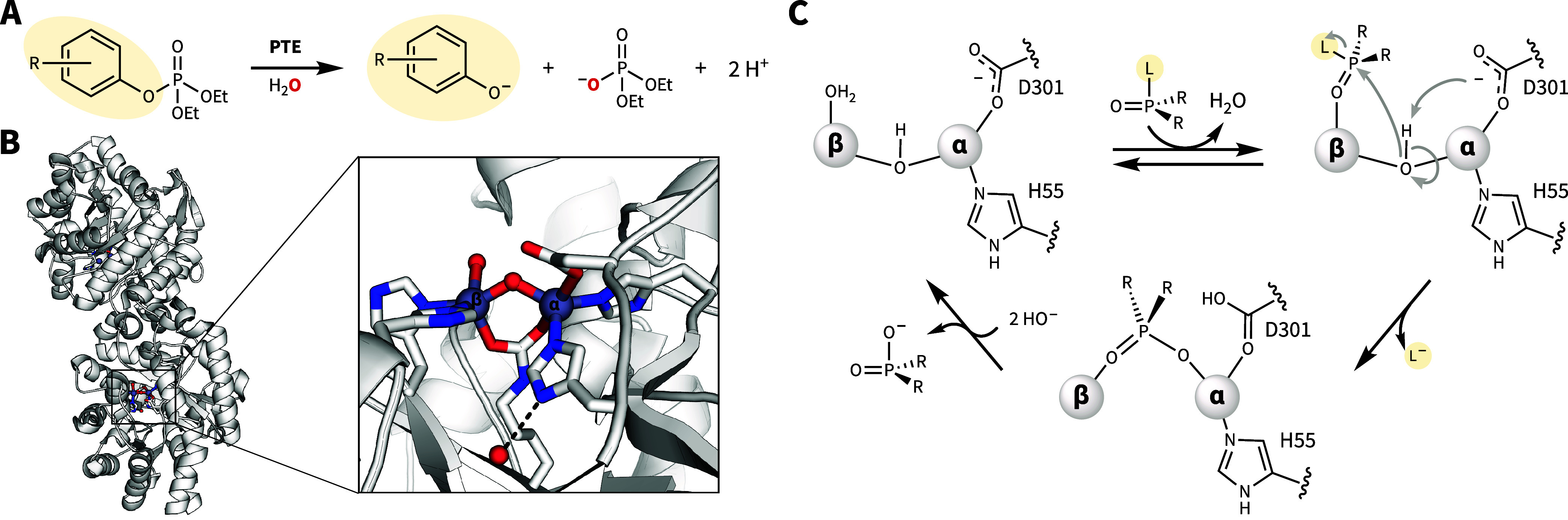
General function and structure of PTE. (A) General PTE catalyzed organophosphate hydrolysis reaction scheme for paraoxon analogues. (B) Dimeric structure and primary coordination sphere of dPTE, PDB 1HZY. (C) Proposed PTE mechanism.

The metal-binding site of dPTE has been probed in great detail through studying metal-based effects and site-directed mutagenesis of the secondary coordination sphere ligands.^15–17^ With respect to metalation, this enzyme is found to contain zinc as primary cofactor but exhibits promiscuity toward Co^2+^, Ni^2+^, Cd^2+^, and Mn^2+^.^8,15^ Leveraging this promiscuity has enabled thorough examination of the role of the metal in the catalytic p*K*_a_. Additionally, within the secondary coordination sphere, substitution of these residues has been used to identify residues critical for catalysis and engineer dPTE for enhanced performance.^18,19^ Mutations of primary coordination sphere amino acids are often more challenging to assess owing to the loss of metal binding. It has been shown that individual substitution of each of the metal-coordinating histidine residues with asparagine has significant deleterious outcomes. The most detrimental impact was reported for H55 (see Methods section for residue numbering), which decreased catalytic efficiency by three to 5 orders of magnitude and impaired metal binding. Comparatively, replacement of the other coordinating histidine residues diminished the activities by one to 2 orders of magnitude. In all cases, the catalytic p*K*_a_ was increased significantly.^16^ These insights provide information regarding the importance of these primary coordination sphere residues, but they also highlight the challenge traditional mutagenesis faces for studying the role of metal-coordinating histidine residues, which have unique binding properties among the canonical amino acids.

In contrast to traditional mutagenesis approaches, noncanonical amino acids (ncAAs) can be incorporated *in vivo* in a site-specific fashion through genetic code expansion.^20,21^ Most commonly, this is achieved by the introduction of an aminoacyl-tRNA synthetase (aaRS)/tRNA pair that enables the ribosomal incorporation of an ncAA in response to an amber stop codon (TAG/UAG). Recent applications of genetic code expansion have enabled researchers to probe metal-binding ligands by introducing structural histidine analogues into iron and copper-containing proteins, achieving precise control of redox potentials and novel insights into structure–function relationships.^22–26^ Thus, we posited that this technique could be used to study and engineer the classic metallohydrolase, dPTE, for improved hydrolytic performance at low pH. Previous ncAA-based engineering approaches in PTEs have focused on targeting positions in the secondary-coordination sphere to enable control of enantioselectivity and introduce electrostatic repulsion of the negatively charged products, enhancing the product release rate.^27–29^ Herein, we demonstrate that substitution of the most catalytically essential histidine (H55) in dPTE with *N* ^π^-methyl-l-histidine (πMH, see Note 1 regarding nomenclature) not only maintains function but improves catalytic activity at low pH by up to 5-fold. Through detailed mechanistic analysis, we show that this improvement arises from systematic decreases in catalytic p*K*_a_ and enhancement of a low-pH catalytic pathway. Together, these findings provide an additional example of the versatility of ncAA-based enzyme engineering, demonstrate opportunities for tailored metalloenzyme engineering, and describe a more acid-tolerant dPTE variant.

## Methods

### General Instrumentation and Materials

Absorbance measurements were performed using a Tecan (Männedorf, CH) Infinite M Nano+ plate reader. ^1^H NMR spectra were recorded in CDCl_3_ on a Bruker AV-AV-400 (400 MHz), chemical shift δ in ppm relative to solvent signals (δ = 7.26 ppm for CDCl_3_), coupling constants J are given in Hz. ^13^C NMR spectra were recorded in CDCl_3_ on a Bruker AV-AV-400 (101 MHz). Unless stated otherwise, all reagents were obtained from commercial sources and used without further purification. Nutrient media were prepared according to supplier recommendations and autoclaved prior to use. Pure water was obtained from a Barnstead MicroPure ST (Thermo Fisher Scientific Inc., Waltham MA, US) water purification system. Buffers were filtered through a 0.22 μm pore-size filter. pH measurements were conducted using a Mettler Toledo (Greifensee, CH) FiveEasy pH meter. Protein purification was performed on an ÅKTA start FPLC system (cytiva, Marlborough MA, US). Automated flash chromatography was conducted on a Isolera One (Biotage, Uppsala, SE) instrument. All sequencing was conducted by Microsynth (Balgach, CH). Protein and DNA concentrations were determined by absorption using a NanoDrop 2000c (Thermo Fisher Scientific Inc., Waltham MA, US) at 280 and 260 nm, respectively. Working antibiotic concentrations were used as followed: 50 μg/mL kanamycin (Kan) and 10 μg/mL tetracycline (Tet).

### Protein Expression

The dPTE2 variant was previously derived from dPTE (UniProtKB: P0A434) through 19 mutations (Supporting Information).^30^ The residue numbering in the text was based on the *Pseudomonas diminuta* wild-type PTE sequence as published previously.^11^ The term *P. diminuta* is used throughout to provide consistent nomenclature with previous dPTE studies, but we note that the organism was reclassified and named as *Brevundimonas diminuta*.^31^ Proteins were expressed with an N-terminal StrepTagII-SUMO-tag fusion on a pET28-Kan^R^ vector. Chemically competent BL21­(DE3) *Escherichia coli* cells (50 μL) were thawed on ice and subsequently incubated with the desired plasmids (∼50–100 ng) for 30 min on ice. The cells were subjected to a heat-shock (42 °C, 30 s) and placed back on ice for 1 min. The cells were then recovered using SOC media (2% (w/v) tryptone, 0.5% (w/v) yeast extract, 10 mM NaCl, 2.5 mM KCl, 10 mM MgCl_2_, 10 mM MgSO_4_, 20 mM glucose, pH 7) and incubated at 37 °C for 1 h before plating on an LB/agar plate containing the appropriate antibiotics. The plates were stored overnight at 37 °C. The next day, single colonies were picked to inoculate 5 mL 2xYT media supplemented with the appropriate antibiotics and incubated overnight at 37 °C. 2xYT media, supplemented with 2 mM ZnCl_2_ or 0.5 mM CoCl_2_ and appropriate antibiotics, was inoculated with the overnight culture (100-fold dilution) and grown at 37 °C until OD_600_ 0.8–1.0. The cultures were cooled on ice, induced by the addition of 0.2 mM IPTG and incubated for additional 24 h at 25 °C. For πMH incorporation, an engineered aminoacyl-tRNA synthetase and tRNA pair from *Methanogenic archaeon* ISO4-G1 (G1PylRS^MIFAF^/PylT_CUA_^G1^)^32^ was coexpressed to enable incorporation of πMH. These cultures were supplemented with 12 mM πMH (Bachem, Bubendorf, CH) 30–60 min prior to induction. The cells were then harvested by centrifugation (4200 rcf, 15 min) and the supernatant was discarded. The pellets were frozen at −80 °C until further use.

### Protein Purification

Frozen cell pellets were thawed on ice and resuspended in lysis buffer (50 mM Tris, 150 mM NaCl, 2 mM MgCl_2_, 0.5 mM CaCl_2_, 1–2 mg/mL lysozyme, 1 μL/mL DNaseI, 2 mM ZnCl_2_ or CoCl_2_, pH 8) and incubated for 2–4 h at room temperature. The cell debris was removed by centrifugation (7197 rcf, 20 min) and the cell-free extract was filtered through a 0.4 μM pore-size filter, before loading onto a 5 mL StrepTactinXT column equilibrated in wash buffer (50 mM Tris, 150 mM NaCl, pH 8) at a flow rate of 0.5 mL/min. The resin was washed with 10 CV of wash buffer at a flow rate of 5 mL/min and subsequently eluted with elution buffer (50 mM Tris, 150 mM NaCl, 50 mM biotin, pH 8) with a flow rate of 0.5 mL/min. Fractions containing the desired protein were combined and concentrated. To cleave the N-terminal StrepTagII-SUMO-tag, StrepTagII-SuperTEV (1:30, protease:protein mass ratio) and DTT (1 mM) were added and incubated overnight at 4 °C. Subsequently a desalting column was used to remove biotin and DTT before removing the SuperTEV and cleaved N-terminus with the StrepTactinXT column, collecting the flow-through with the desired protein at a flow rate of 0.5 mL/min. The purified proteins were stored in Tris (50 mM, pH 8), NaCl (150 mM), ZnCl_2_ or CoCl_2_ (0.1 mM) at 4 °C.

### Activity Assays

A 2× enzyme solution (100 μL, 2 nM in 40 mM each acetate/MES/HEPES/Tris, 100 mM NaCl, 1 mg/mL BSA) was added to a clear 96-well plate charged with reaction buffer (50 μL, 40 mM each acetate/MES/HEPES/Tris, 100 mM NaCl, 1 mg/mL BSA). Subsequently a 4× substrate solution (50 μL in 40 mM each acetate/MES/HEPES/Tris, 100 mM NaCl, 20% ACN) was added and the release of the corresponding phenolate product was monitored using a Tecan Infinite M Nano+ microplate reader set to 25 °C with measurement intervals of 10 s (see Supporting Data for wavelengths and calibration curves). Deuterium solvent isotope experiments were conducted with the same buffer compositions prepared with >95% D_2_O. The pH was adjusted with NaOD or DCl. Where 4 < pH, *D* < 8, no correction was applied to the pH measurement. For pH, *D* > 8, +0.42 was added to the pH meter readout, according to literature.^33,34^

### Data Analysis

All activity data were analyzed with GraphPad Prism9 by fitting to the appropriate equations. Statistical analyses (ANOVA, *F*-test, AIC, etc.) were performed using the built-in functions in GraphPad Prism9. When comparing models for data, visual inspection of the fit (overlay of the predicted and experimental), analysis of the residuals (shape, distribution, and homoscedasticity), and the model probability based on the AIC were all used to decide between models. The pH profiles were modeled with either a simple single ionization model (eq 1) a more complex model in which the enzyme contains two active ionization states interconverting through the ionization of a single species (eq 2), and a model with two ionizable species in which deprotonation of each improves catalysis (eq 3). In these equations, *y* is the observed catalytic parameter, and *c* represents the intrinsic catalytic rate constant.

1 $$\log\left(y\right)=\log\left(\frac{c}{\left(1+10^{\left({pK}_{a}-\mathrm{pH}\right)}\right)}\right)$$

2 $$\log\left(y\right)=\log\left(k_{\mathrm{cat},\mathrm{ESH}+}+\frac{\left(k_{\mathrm{cat},\mathrm{ES}}-k_{\mathrm{cat},\mathrm{ESH}+}\right)}{\left(1+10^{\left({pK}_{a}-\mathrm{pH}\right)}\right)}\right)$$

3 $$\log\left(y\right)=\log\left(\frac{c}{(1+10^{\left({pK}_{a1}-\mathrm{pH}\right)})\left(1+10^{\left({pK}_{a2}-\mathrm{pH}\right)}\right)}\right)$$

The values of the Michaelis–Menten parameters were determined from a fit to eq 4, where *v* is the initial velocity, *V*_max_ is the maximum velocity, *K*_m_ is the Michaelis–Menten constant, and *A* is the substrate concentration.

4 $$v=\frac{V_{\max}\times A}{\left(K_{m}+A\right)}$$

The rate contribution of the ESH+ pathway at elevated pH was determined by eq 5, where the fraction of ESH+ present is estimated by the catalytic p*K*_a_.

5 $$\mathrm{rate}\;\mathrm{contribution}\;\left(\mathrm{ESH}+\right)=\frac{\left({{\mathrm{fraction}}_{\mathrm{pH},\mathrm{ESH}+}\cdot k}_{\mathrm{cat},\mathrm{ESH}+}\right)}{\left({{\mathrm{fraction}}_{\mathrm{pH},\mathrm{ESH}+}\cdot k}_{\mathrm{cat},\mathrm{ESH}+}+{{\mathrm{fraction}}_{\mathrm{ES}}\cdot k}_{\mathrm{cat},\mathrm{ES}}\right)}$$

The data of the Brønsted plots was fitted to the minimal kinetic model using eqs 6 and 7 combined with the Brønsted catalysis eq 8.

6 $$\log\left(k_{\mathrm{cat}}\right)=\log\left(\frac{k_{3}k_{5}}{k_{3}+k_{5}}\right)$$

7 $$\log\left(\frac{k_{\mathrm{cat}}}{K_{M}}\right)=\log\left(\frac{k_{1}k_{3}}{k_{2}+k_{3}}\right)$$

8 $${\log(k}_{3})=\beta {pK}_{a}^{\mathrm{LG}}+C$$

### High-Resolution LC-MS Analysis

Samples were diluted with 1% TFA, passed through the AttractFiltra RC Micro Spin column, and transferred to autosampler vials for LC/MS. Samples were injected into a BioResolve Premier RP-mAb 2.7 μm, 2.1 mm × 20 mm, 450 Å (Waters, USA) column. For desalting respectively separation on an Acquity UPLC station, a gradient buffer A (0.1% DFA in water)/buffer B (0.1% DFA in AN/75% 2-PrOH) at a flow rate 200 μL/min at 60 °C over 30 min was applied. The analysis was performed on a Synapt G2-Si mass spectrometer directly coupled to the UPLC station. Mass spectra were acquired in the positive-ion mode by scanning the *m*/*z* range from 400 to 5000 Da with a scan duration of 1 s and an interscan delay of 0.1 s. The spray voltage was set to 3 kV, the cone voltage to 50 V, and the source temperature to 100 °C. The data were recorded with the MassLynx 4.2 Software (both Waters, UK). The recorded *m*/*z* data of single peaks or their slices were deconvoluted into mass spectra by applying the maximum entropy algorithm MaxEnt1 (MaxLynx) with a resolution of the output mass 0.5 Da/channel and Uniform Gaussian Damage Model at the half height of 0.5 Da.

### High-Resolution LC-MS/MS Analysis

Proteins were reduced and alkylated by adding tris­(2-carboxyethyl)­phosphine and chloroacetamide to a final concentration of 5 mM and 15 mM, respectively. The samples were incubated for 30 min at 30 °C; 700 rpm and light-protected. Digestion was done in buffered trypsin solution at pH 8 (10 mM Tris/2 mM CaCl_2_). Samples were enzymatically digested and dried. The digested samples were dissolved in aqueous 3% Acetonitrile with 0.1% formic acid, and the peptide concentration was estimated with the Lunatic UV/vis absorbance spectrometer (Unchained Lab). Peptides were separated on a M-class UPLC and analyzed on a Orbitrap mass spectrometer (Thermo).

### ICP-MS

Metal standards were acquired from Merck (Darmstadt, DE). Protein samples (100 μL) were diluted in conc. trace-metal grade HNO_3_ (400 μL) and spiked with 1000 ppb Ga as internal standard. The samples were incubated at 25 °C overnight and subsequently heated to 70 °C for 3–7 h. The samples were centrifuged briefly (20,000 rcf, 10 min) and diluted with pure H_2_O to a final volume of 3 mL. Control samples without protein were prepared accordingly. The samples were then measured on an Agilent 8800 triple-quadrupole ICP-MS equipped with a standard x-lens setting, nickel cones and a “micro-mist” quartz nebulizer. The feed was 0.1 mL/min, the RF power 1550 W. Tune settings were based on the Agilent General Purpose method and only slightly modified by an autotune procedure using an Agilent 1 ppb tuning solution containing Li, Y, Ce and Tl. Values are reported as the average of 30 sweeps × 3 replicates.

### Crystallography

Crystals were grown using the sitting-drop vapor diffusion method in 200 nL drop volumes. In an initial screen, zinc-substituted dPTE2-H55 (40 mg/mL) and dPTE2-H55πMH (24 mg/mL) solutions in HEPES (20 mM, pH 8) were screened against commercial BCS (MD1–104) and JCSG+ (MD1–37, Molecular Dimensions) screens at 4 °C. An initial hit was found for both variants in 20% (w/v) PEG8000, 100 mM Tris (pH 8.5), 200 mM MgCl_2_ within 2 days. These crystals were used to create a seedstock using HR2–320 PTFE seed beads (Hampton Research) according to the manufacturer’s instructions. The seedstocks (20 nL) were used to seed a custom screen based on previously published conditions. Crystals grew within 1 week at 4 °C. dPTE2-H55 crystallized in 18% (w/v) PEG8000, 100 mM Bicine (pH 8.3), 1 M LiCl, and dPTE2-H55πMH in 15% (w/v) PEG8000, 100 mM Bicine (pH 8.0), 100 mM NaCl, at 4 °C. Crystals were transferred to the reservoir solution containing 20% (v/v) ethylene glycol as cryoprotectant before flash freezing in liquid nitrogen.

Data were collected at ESRF ID30A-3/MASSIF-3 Beamline at 0.9677 Å. The data was processed using the autoPROC pipeline.^35–40^ The data of dPTE2-H55 and dPTE2-H55πMH could be merged and scaled to 1.55 Å and 1.69 Å, respectively, and were solved by molecular replacement with an AlphaFold3 ^41^ model of the dPTE2 wild-type structure and iterative refinement in coot^42^ and phenix.refine.^43^ The H55 variant crystallized in the *P*2_1_2_1_2_1_ space group with two molecules in the asymmetric unit and 47% solvent content. The πMH variant crystallized in the *C*1_2_1 space group with six molecules in the asymmetric unit and 48% solvent content. dPTE2-H55 could be refined to an *R*_work_/*R*_free_ 0.1775/0.1872 and a Molprobity score of 0.96. dPTE2-H55πMH could be refined to an *R*_work_/*R*_free_ 0.2039/0.2164 and a Molprobity score of 1.18.

The PDB models have been deposited in the PDB as 9TI1 (H55) and 9TI2 (H55πMH), respectively. In the PDB deposition, πMH was termed MHS and the carboxylated lysine was termed KCX based on previous PDB entries. Crystallographic details are provided in Supporting Table 37.

## Results and Discussion

### The Highly Essential, α-Metal-Coordinating H55 Can Be Efficiently Replaced with πMH

To facilitate expression and ensure stability, we selected a previously described engineered variant of dPTE containing 19 stabilizing mutations outside of the active site (dPTE2).^30^ As a target for genetic code expansion, residue H55 was selected for its essentiality in catalysis^16^ and its lack of hydrogen bonding with other amino acids. Instead of hydrogen bonding with other amino acids, the noncoordinating πN of H55 forms a hydrogen bond with an ordered water molecule. Thus, we anticipated that modification of this residue was less likely to disrupt protein folding. Several histidine mimics with varying conjugate acid p*K*_a_s and σ-donor properties can be genetically incorporated.^44^ Thus, we screened six histidine-like ncAAs with varying conjugate acid p*K*_a_s to identify an ncAA that could replace H55 and retain high activity (Supporting Figure 1). From the ncAAs screened, five have significantly lower conjugate acid p*K*_a_s than histidine with predicted p*K*_a_Hs ranging from 2.5 to 4.7 for the free amino acid;^44^ only one has a slightly higher conjugate acid p*K*_a_s than histidine: πMH (predicted aqueous p*K*_a_H of 6.7 πMH vs 6.5 His). Notably, the predicted conjugate acid p*K*_a_s were based on an aqueous environment, but protein microenvironments can significantly alter p*K*_a_s, particularly in cases where hydrogen bonding and metal binding are involved, such as for H55. Nonetheless, we found that only the incorporation of πMH resulted in appreciable activity in our screens. All other ncAAs tested exhibited at least 9-fold reduction in *k*_cat_. Metal content analysis for the variants demonstrated that the correct metal-to-monomer stoichiometry (2:1) was retained for four of the five variants with low conjugate acid p*K*_a_s, suggesting that the low activity is likely a result of incompetent active sites rather than loss of metal coordination (Supporting Figure 1). The success of πMH suggests that its parameters are within a narrow tolerance for maintaining both metal coordination and catalytic function. This selectivity underscores both the challenge and opportunity in engineering primary metal coordination spheres with ncAAs. Based on these results, we elected to focus on comparative analysis of dPTE2-H55 and dPTE2-H55πMH (Figure 2A).

**Figure 2:**
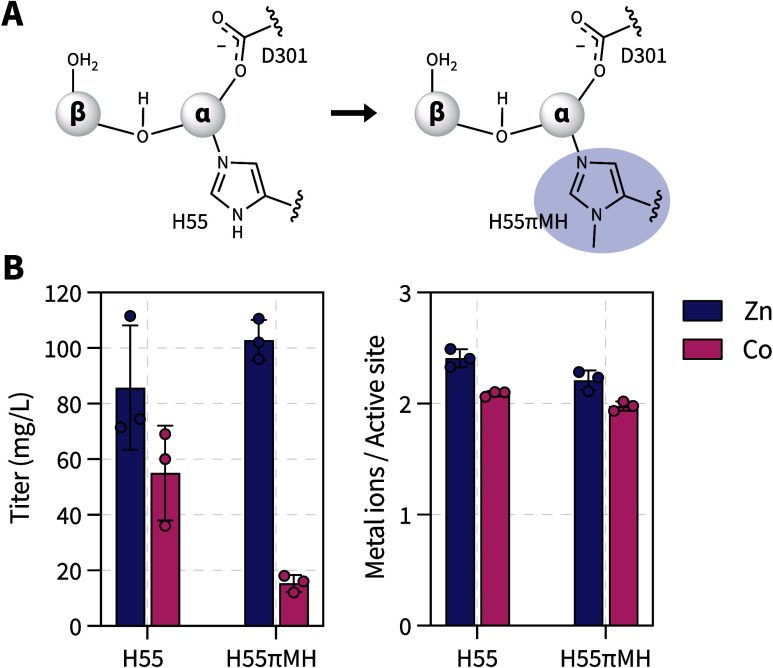
Substitution of dPTE2-H55 with πMH. (A) Replacement of the native histidine with *N* ^π^-methyl-l-histidine (πMH). (B) Expression titers after purification of the dPTE2-H55 and corresponding πMH variants substituted with either Zn^2+^ or Co^2+^(left). Metal quantification by ICP-MS (right). Bars represent the mean of three biological replicates with error bars representing the standard deviation. Individual data points are shown as dots.

For both dPTE2-H55 and dPTE2-H55πMH, protein production in the presence of exogenous zinc resulted in high titers between approximately 80–110 mg/L (Figure 2B). The protein identity was confirmed by LC-MS of the intact proteins, and the successful incorporation of the πMH at the target position was additionally validated by LC-MS/MS of the trypsinized protein (Supporting Figure 2). The metal loading was approximately two zinc ions per monomer as determined by ICP-MS (Figure 2B and Supporting Figure 3). To probe the effect of the metal ion, we also produced these proteins in the presence of exogenous cobalt. Supplementation of cobalt in the growth media led to a decrease in the overall cell viability and expression levels, which was more pronounced for the πMH variant, likely owing to combined toxicity and metabolic stress. Despite the lower yields, both LC-MS and LC-MS/MS confirmed high purity and incorporation fidelity for πMH for the cobalt-based expression (Supporting Figure 2). ICP-MS analysis also confirmed the expected two cobalt ions per monomer (Figure 2B and Supporting Figure 3). These results indicate that substitution of the highly essential, α-coordinating H55 with πMH can be achieved with relatively high protein yield and efficient coordination of either zinc or cobalt.

### H55πMH Variants are Highly Active

To assess the catalytic activities of our enzyme variants, we employed an established reaction with the classical substrate paraoxon (III) and derivatives thereof, facilitating the straightforward readout of conversion by monitoring the absorbance of the generated phenolate product (Figure 1A). These substrates were chosen based on the p*K*_a_ values of the phenolic leaving group (p*K*_a_^LG^, Figure 3).^45,46^ The catalytic activity was measured at slightly acidic pH 6. Under these conditions, no activity could be confidently observed for substrate V, which has the highest p*K*_a_^LG^ and the smallest change in absorbance between the substrate and products. For substrates I, II, and III, which have low p*K*_a_^LG^, substitution of H55 with πMH improved *k*_cat_ values by 1.4- to 1.9-fold and *k*_cat_/*K*_m_ values by 1.4- to 2.4-fold. The changes in *k*_cat_ were similar for both the zinc- and cobalt-containing proteins, but the changes in *k*_cat_/*K*_m_ were more pronounced when the active site contained zinc rather than cobalt. These low p*K*_a_^LG^ are often referred to as “fast substrates” because their chemical step(s) are fast and not rate-limiting. The rate-limiting steps for these substrates are typically attributed to steps after P–O bond cleavage, possibly including release of product or regeneration of the active catalyst.^15,45,46^ In contrast, substrates with high p*K*_a_^LG^ are referred to as “slow substrates” and are limited by an early chemical step(s) that is linearly dependent on p*K*_a_^LG^, often reported to be cleavage of the P–O bond.^45,46^ With substrate IV, the activities of all variants were significantly lower than observed for the low p*K*_a_^LG^ substrates at pH 6. The H55πMH substitution resulted in a 1.2- and 1.4-fold improvement in the *k*_cat_ and *k*_cat_/*K*_m_, respectively, but only for the zinc supplemented variants. No improvements were observed for the cobalt containing variants. Based on these results, we concluded that substitution of H55 with πMH enables higher activity at acidic pH for substrates with low leaving group p*K*_a_s, but the magnitude of these improvements differs between metals and *k*_cat_ and *k*_cat_/*K*_m_.

**Figure 3:**
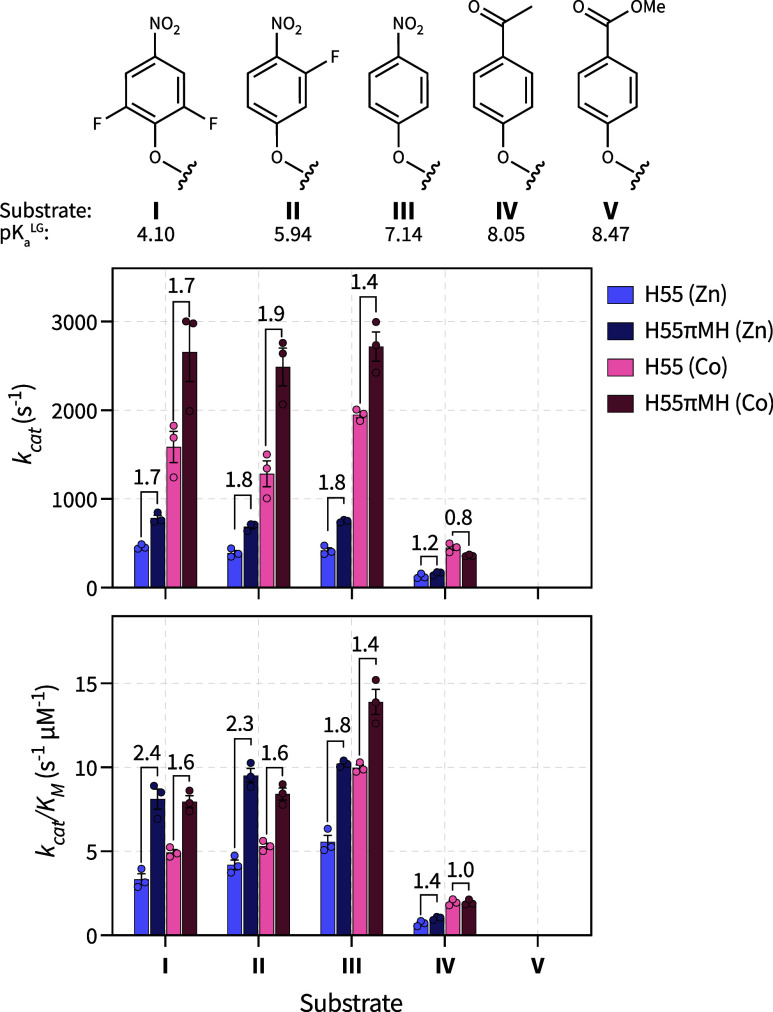
Comparison of catalytic activity at slightly acidic pH. Chemical structures of the substrate leaving groups and their corresponding p*K*_a_^LG^. Michaelis–Menten parameters for these substrates at pH 6 with indicated fold change of the πMH variants. Bars represent the mean of three biological replicates with error bars representing the standard deviation. Individual data points are shown as dots.

### H55πMH Variants Consistently Exhibit Lower Catalytic p*K*_a_

To further understand the changes imposed by H55πMH, we measured the Michaelis–Menten kinetics across a wide pH range: 4 to 8.5 for substrates I–IV (Supporting Figures 4–8). Substrate V could not be rigorously assessed at neutral to low pH, owing to low extinction coefficient changes between the substrate and products. For completeness, the data at high pH are provided in Supporting Figures 9 and 10. The extracted Michaelis–Menten parameters were plotted as a function of pH (Figure 4A). With substrates I–III, no significant changes were observed upon substitution of H55 with πMH at higher pH values. Notably, the values for *k*_cat_/*K*_m_ at high pH were not affected by metal identity, substrate p*K*_a_^LG^, or H55/H55πMH, indicative of diffusion-limited kinetics. In contrast, with decreasing pH, the *k*_cat_ and *k*_cat_/*K*_m_ are significantly improved by the H55πMH substitution, increasing up to 5-fold. These results suggest that substitution of H55 with πMH is nondetrimental at high pH and advantageous at low pH for substrates with low p*K*_a_^LG^. In contrast, for substrate IV at high pH, the πMH substitution significantly decreases the *k*_cat_ and *k*_cat_/*K*_m_, with the largest effect being a 2-fold reduction in activity. At low pH with substrate IV, the πMH variant equalizes or slightly exceeds the activity of the H55 variant in the case of the cobalt and zinc variants, respectively. These results suggest that substitution of H55 with πMH is detrimental to the observed activity at high pH for substrates with higher p*K*_a_^LG^s. Collectively, the data reveal a significant substrate-dependent effect and a smaller metal-dependent effect with respect to the changes exerted by the πMH substitution.

**Figure 4:**
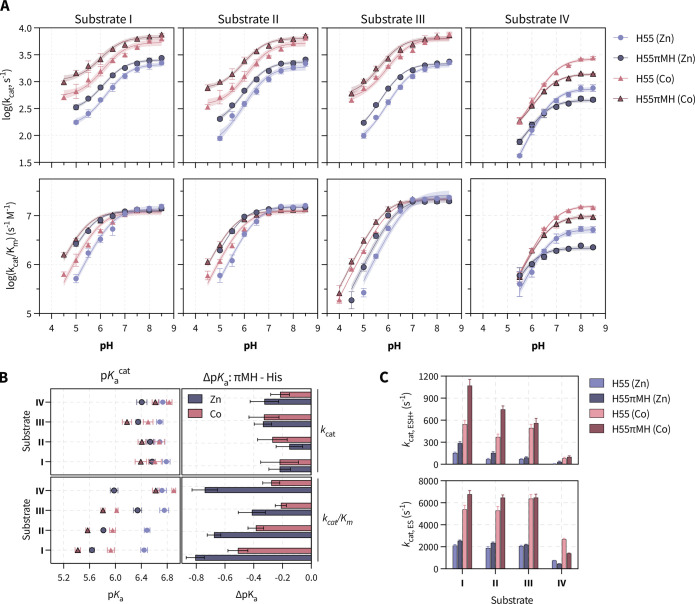
pH profile analysis of the H55 and H55πMH variants. (A) pH-rate profiles for substrates with varying p*K*_a_ of the phenolic leaving group. Data points represent the mean of three biological replicates; the error bars represent standard deviation; and the shading indicates the 95% CI of the p*K*_a_ fit to eq 2 (*k*_cat_) or eq 1 (*k*_cat_/*K*_m_). (B) From the pH profile analysis, the observed catalytic p*K*_a_s were extracted and the changes upon substituting histidine with πMH were determined. The bars represent the mean of three biological replicates, and the error bars represent the standard deviation. (C) For the *k*_cat_ model, the rate constants for the lower activity protonated state (top, ESH+) and for the higher activity deprotonated state (bottom, ES) were extracted.

Previous work indicates that the α-metal is the primary contributor to the apparent catalytic p*K*_a_ (p*K*_a_^cat^).^11,15^ Based on the observed effects of the πMH over the pH range, we anticipated that this substitution might significantly lower the p*K*_a_^cat^. The resulting pH profiles were modeled with a simple single ionization model (eq 1), a more complex model in which the enzyme contains two active ionization states interconverting through the ionization of a single species (eq 2), and a model with two ionizable species in which deprotonation of each improves catalysis (eq 3). The best models were selected by comparing agreement between the data and the model, residuals, and the Akaike information criterion (AIC).^47^

The pH profiles for *k*_cat_ were best modeled by eq 2, with p*K*_a_^cat^ values between 6.0 and 6.8 (Figure 4A,B and Supporting Figure 11). The pH profiles for *k*_cat_/*K*_m_ were best modeled by eq 1, with p*K*_a_^cat^ values varying more widely between 5.3 and 7.0 (Figure 4A,B). Such differences in the p*K*_a_^cat^ values of these two parameters have been reported for dPTE but the source of the difference is not defined.^15^ In a simple model, the p*K*_a_^cat^ values for *k*_cat_ and *k*_cat_/*K*_m_ reflect ionization of ES complex and the free enzyme, respectively.^48^ Thus, the source of these differences between H55 and H55πMH could reflect differences in the ionizable species of the free enzyme and the ES complex or changes in the rate-limiting step or general mechanism across the pH range. The difference between the p*K*_a_^cat^ values derived from the *k*_cat_ and *k*_cat_/*K*_m_ is less than 1. Thus, it is possible that the ionizable groups related to *k*_cat_ and *k*_cat_/*K*_m_ are the same species, and that substrate binding alters the p*K*_a_ of the ionizable species. For PTEs, substantial kinetic evidence at high pH suggests that the ionizable species is the bridging hydroxide.^12,15,49^ Binding of the substrate to the β-metal would likely modulate the p*K*_a_ of this bridging hydroxide, suggesting a possible explanation for the difference in the p*K*_a_^cat^ values from these two parameters. The model for the p*K*_a_^cat^ corresponding to *k*_cat_ suggests that there are two distinct mechanisms with catalytically competent ES states, generated by different protonation states of a single ionizable species. The *k*_cat_ values corresponding to those different catalytic states were extracted (termed *k*_cat,ESH+_ and *k*_cat,ES_, Figure 4C). The protonated ESH+ complex has a significantly lower rate constant than the ES complex. If the bridging hydroxide is protonated to form a water molecule, direct nucleophilic attack would be significantly impaired. However, PTE-catalyzed hydrolysis might still be possible through compensatory hydrogen bonding interactions in the active site.

With respect to πMH, we observed that the H55 substitution with πMH decreased the p*K*_a_^cat^ values in all cases (Figure 4B). The changes in the p*K*_a_^cat^ related to *k*_cat_/*K*_m_ were generally larger in magnitude than those related to *k*_cat_. These results are consistent with our observation that H55πMH improves *k*_cat_/*K*_m_ more than *k*_cat_. Moreover, *k*_cat,ESH+_ and also *k*_cat,ES_ increased upon substitution of H55 with πMH. The improvements in both *k*_cat,ESH+_ and *k*_cat,ES_ generally increased with decreasing p*K*_a_^LG^. However, the improvements in *k*_cat,ESH+_ were significantly larger than the improvements in *k*_cat,ES_, suggesting that substitution of H55 with πMH improves the catalytic properties of the protonated pathway to a greater extent. These results suggests that πMH substitution is generally beneficial for catalysis at low pH with differing effects on the two operative mechanisms.

To gain further insights into these observations, we examined the pH profile in >95% D_2_O for substrate III at varying pH to determine the kinetic solvent isotope effect (KSIE, Supporting Figures 12–13). At high pH, we observed a KSIE of *k*_cat_ around 2–2.6 and a KSIE of *k*_cat_/*K*_m_ around 1, consistent with previous reports.^15^ Over the extended pH range, the most prominent observation is an increase in the KSIE of *k*_cat_ at lower pH; the onset of which appeared to be near the p*K*_a_^cat^ values. These observations provide further support for a change between two catalytically competent ES states. The change results in a different rate constant with a higher KSIE which could result from either proton transfer becoming more rate-limiting or altering commitment factors prior to a rate-limiting proton transfer step. Interestingly, there was also a pH-dependent change in the KSIE of *k*_cat_/*K*_m_, but only for the zinc-containing enzymes, suggesting that the proton transfer may be differentially regulated in the zinc and cobalt systems, with zinc involving proton transfer in formation of the ES complex and cobalt involving proton transfer in the rate-limiting step or alteration of commitment factors. This observation is also supported by a greater Δp*K*_a_ (H_2_O–D_2_O) of *k*_cat_ for cobalt than for zinc (Supporting Figure 14).^50^

Upon substitution of H55 with πMH, a small decrease in the KSIEs associated with *k*_cat_ can be observed. With the exception of the cobalt-containing variant with substrate III, the changes were typically within the error (Supporting Figure 14). Collectively, these results suggest that there are two catalytically competent ES complexes that differ by the protonation state of a single ionizable species—likely the bridging hydroxide (Supporting Figure 15). The protonated ESH+ state displays significantly lower activity than the ES state. Substitution of H55 with πMH increases the rate constant for catalysis of the ESH+ complex and decreases the p*K*_a_^cat^, extending the operating range of the ES complex.

### Brønsted Analysis

For acid catalysis, including Lewis acid catalysis, a Brønsted plot—describing the reaction rate as a function of substrate ionization constant—can be used to uncover mechanistic features. Thus, we performed a Brønsted analysis with substrates I–V, which have varying p*K*_a_^LG^. Because substrate V could not be assessed below pH 7.5, the Brønsted analysis was limited to pH 7.5–8.5. Analysis of the rate constants and p*K*_a_^cat^ values for the ES and ESH+ pathways indicates that in this pH range, the ESH+ pathway contributes less than 2% to the observed catalytic rate (eq 5). Thus, a simplified mechanism only accounting for the ES pathway was applied (Figure 5A). The curves were nonlinear with a plateau at low p*K*_a_^LG^ (Figure 5B and Supporting Figure 16) in agreement with previous observations, identifying “slow” substrates that are rate-limited by an early chemical step (high p*K*_a_^LG^) and “fast” substrates that exhibit rapid chemistry and are limited by step(s) after P–O bond cleavage (low p*K*_a_^LG^).^45,46^

**Figure 5:**
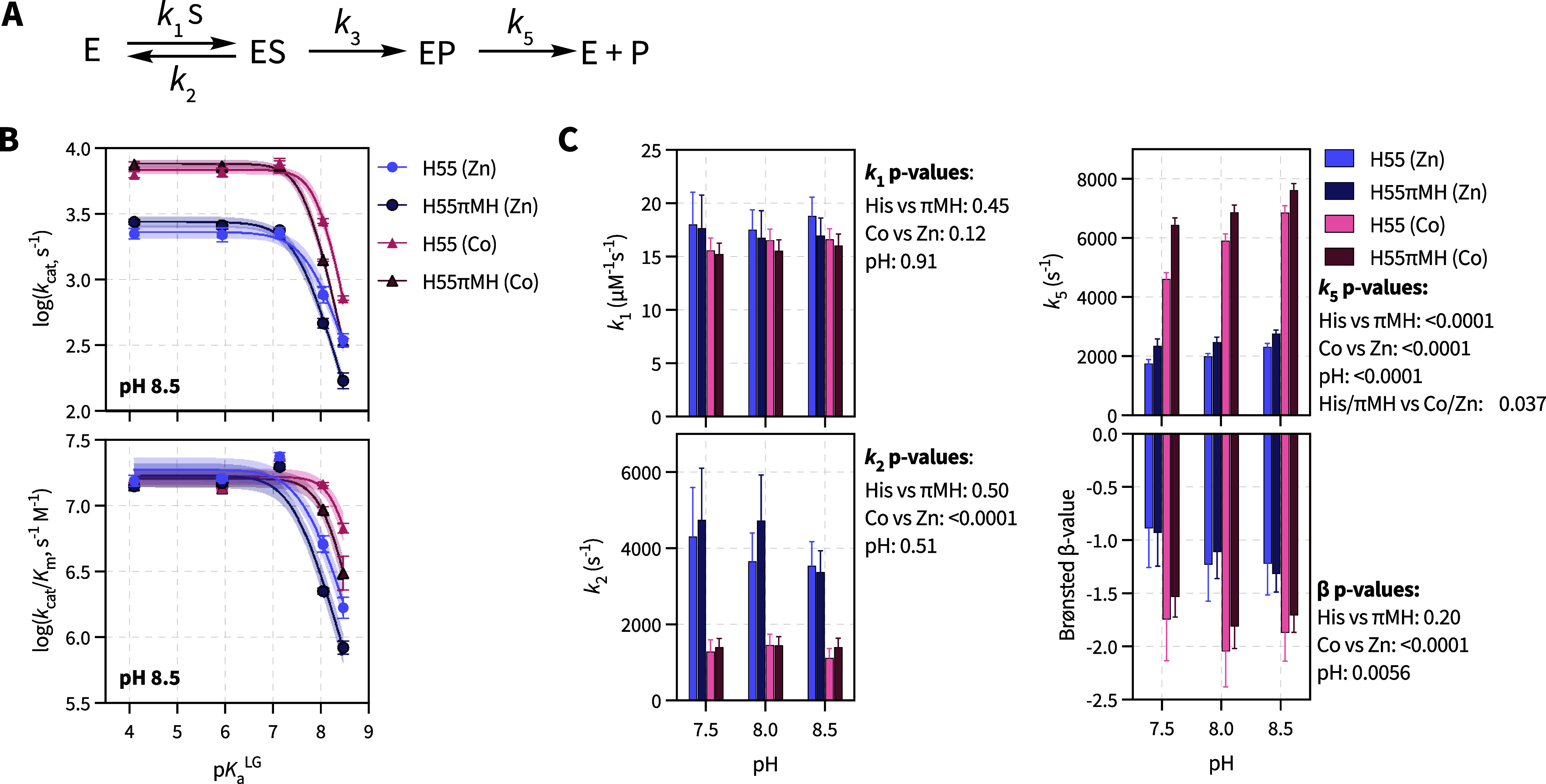
Brønsted analysis of the enzymatic hydrolysis of substrates I–V for the H55 and H55πMH variants. (A) Classical reaction pathway for PTE catalyzed organophosphate hydrolysis. (B) Brønsted plot for the dependence of log­(*k*_cat_) (top) and log­(*k*_cat_/*K*_m_) (bottom) on the p*K*_a_^LG^ at pH 8.5. Data points represent the mean of three biological replicates; the error bars represent standard deviation; and the shading indicates the 95% CI of the model fit to the equations: eq 6 (*k*_cat_) or eq 7 (*k*_cat_/*K*_m_) using eq 8 (Brønsted catalysis). (C) Individual rate constants and Brønsted β-parameter, derived from the fit to the minimal kinetic model. Three-way ANOVA with Tukey posthoc testing was used to determine statistically significant factors.

The Brønsted-plots were well described by the minimal kinetic model^45,46^ classically used for PTE catalyzed organophosphate hydrolysis (Figure 5A). This model was used to deconvolute the observed kinetic parameters into the individual rate constants for interaction with the substrate (*k*_1_ and *k*_2_), the chemical hydrolysis step (*k*_3_), and the release of the product and regeneration of the enzyme (*k*_5_). Additionally, the Brønsted-parameters (β-values) provide insights into the nature of the transition state (Figure 5C). We performed a three-way ANOVA with Tukey posthoc testing to evaluate which factors significantly impacted the observed differences.^51–54^ For *k*_1_, no investigated parameter displayed any significant change. In contrast, *k*_2_ is strongly affected by the identity of the metal, suggesting that the cobalt ES complex is less prone to substrate release than its zinc counterpart. *k*_5_ exhibits a dependence on metal identity, pH, and H55 vs H55πMH. Additionally, there is an apparent interaction factor between metal identity and H55/H55πMH. Substitution of H55 with πMH increases the rate of *k*_5_ in a pH-dependent manner, which points toward a correlation between the apparent p*K*_a_^cat^ and *k*_5_, either directly or indirectly through commitment factors. The derived *k*_5_ values are very similar to the *k*_cat,ES_ found for substrates I–III (Figure 4C), suggesting that this *k*_5_ step is significantly rate-limiting for the ES catalytic pathway. In this simplified model, *k*_5_ corresponds to irreversible steps following the P–O bond cleavage. These steps include the release of the phenolic leaving group, the release of the phosphate product, and the regeneration of the active enzyme state, which involves the formation of the bridging hydroxide species. Lastly, the β-values for zinc and cobalt were −1.12 ± 0.17 and −1.79 ± 0.17, respectively, consistent with previous reports and indicative of a late, product-like, transition state with a significant charge transferred to the leaving group phenol.^45^ These results suggest a nearly completely broken P–O bond in the transition-state. Notably, many of the β-values were less than −1.0, particularly in the case of cobalt. Values below −1 are commonly interpreted evidence of strong electronic reorganization and protein microenvironment effects in the transition state, making the reaction more sensitive to negative charge accumulation than a simple equilibrium ionization.^55^ No significant change in the β-values was observed upon substitution of H55 for H55πMH substitution at pH 7.5–8.5, suggesting that the πMH substitution does not fundamentally alter the nature of the transition state or charge distribution in the rate-limiting step under these alkaline conditions. The improvements in *k*_5_, suggest that at alkaline pH, the πMH substitution primarily affects product release or enzyme regeneration rather than the chemical step itself for fast substrates. However, we cannot rule out changes in β-values at acidic pH, where the πMH variant shows the greatest improvement. Notably, we expect that a Brønsted analysis at low pH would be more revealing of the positive effects exerted by πMH, but unfortunately, we were not able to fit the model stably without the substrate V, which was not possible to assay at low pH.

### The Methyl Group Displaces an Ordered Water Molecule

To understand the atomistic changes upon H55 substitution with πMH, we explored structural characterization. We were able to obtain high-quality crystals only under basic conditions for the zinc-containing H55 and H55πMH enzymes, which diffracted to 1.55 Å and 1.69 Å, respectively. The monomers of the two variants align well with an RMSDs of 0.135–0.142 Å (entire main chain). Additionally, both the H55 and H55πMH variants align with a published wild-type dPTE structure^11^ with RMSDs of 0.159–0.179 Å and 0.200–0.214 Å, respectively. From these crystal structures, minimal changes in the coordination chemistry can be systematically identified (Figure 6). The most evident difference is the displacement of the water molecule, hydrogen-bonding to H55, by the methyl group in the H55πMH variant. Although we observed differences in the Zn–Zn, Zn­(β)−μO, and μO–O_Asp301_ bonds, the differences were not statistically significantly different when considering each of the monomers in the unit cell. Additionally, the Zn­(α)–H55πMH distance is comparable to the corresponding Zn­(α)–H55 distance. The closest reported structure to the H55 variant (derived from dPTE2) is the wild-type dPTE structure.^11^ The active site and bond lengths observed for both our H55 and H55πMH are also similar to those in the wild-type dPTE structure (Figure 6C).

**Figure 6:**
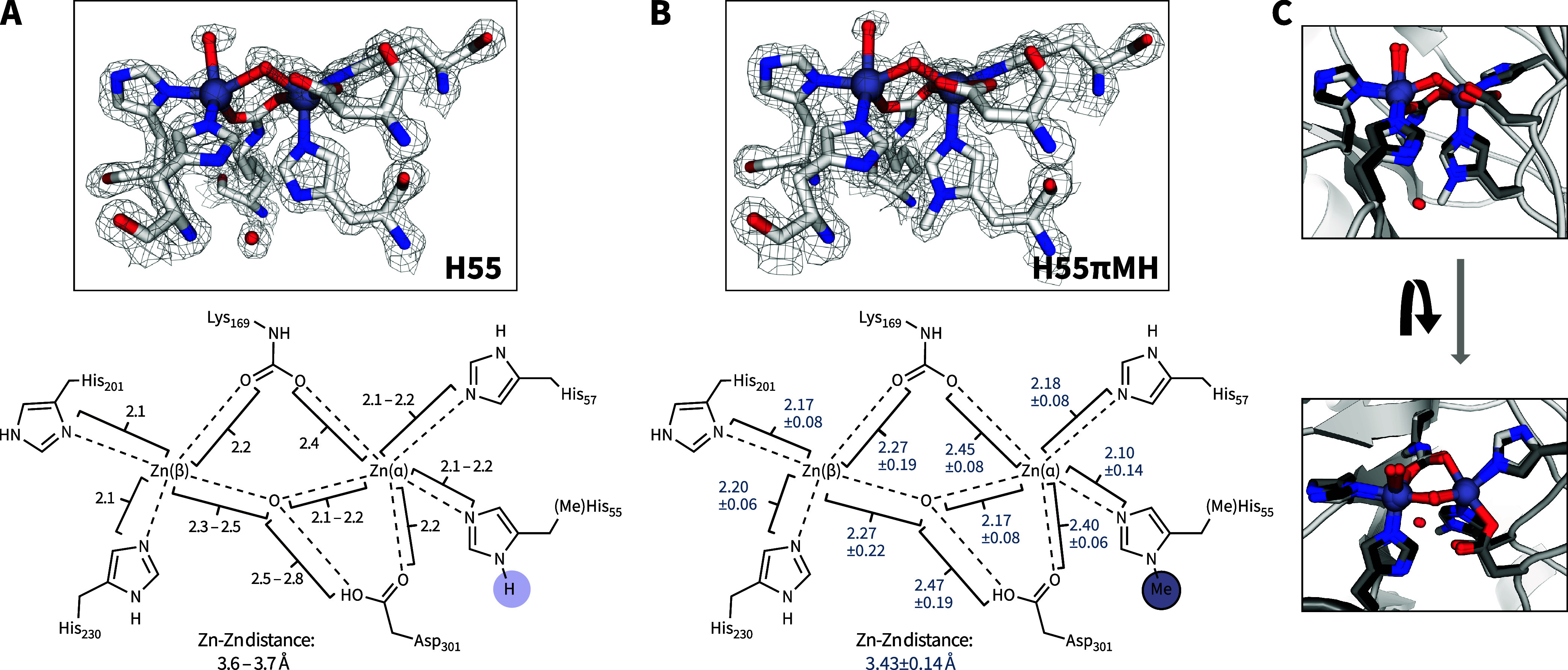
Structural analysis illustrating the electron density—2Fo – Fc electron density map contoured at 2.0σ—at the active sites of dPTE2-H55 (A, top) and dPTE2-H55πMH (B, top), the atomic distances in Å within the active site dPTE2-H55 (A, bottom) and dPTE2-H55πMH (B, bottom), and (C) two orientations with the overlay of dPTE2-H55πMH (white), dPTE2-H55 (gray), and dPTE (PDB 1HZY, black). The zinc ions are shown as gray-blue spheres; the water molecules are shown as red spheres, and the metal ligands are shown as sticks. The atomic distances are given as a range for dPTE2-H55 and the average and standard deviation for dPTE2-H55 πMH, which have six and two monomers in the asymmetric unit, respectively.

The lack of significant differences in the active sites at alkaline pH is consistent with our observation that H55 and H55πMH exhibit similar Michaelis–Menten parameters under these conditions. The functional divergence at acidic pH—where H55πMH shows superior activity—likely arises from pH-dependent changes in coordination geometry, protonation states, or active site dynamics that are not captured in these static, high-pH structures. Unfortunately, we were unable to obtain crystals at acidic pH with the dPTE2 variant. Notably, it has been demonstrated in cobalt-substituted carbonic anhydrase that the pH significantly alters the coordination chemistry.^56^ However, in the only published structures of wild-type dPTE in acidic conditions (pH 6), no significant change in coordination chemistry can be observed,^57^ suggesting that complete rearrangement of the active site, such as a change in coordination number, is unlikely.

## Conclusion

Engineering the primary coordination sphere of metalloenzymes using ncAAs has been leveraged to alter or improve properties of metalloenzymes with iron-heme^22^ and copper^58^ cofactors. The extension of this technique to engineer and study diverse metal sites has significant potential in biochemistry and enzyme engineering but remains underexplored. Herein, we examined ncAA-based engineering with a well-studied proof-of-concept target—an engineered metal-dependent PTE from *P. diminuta* (dPTE2). We selected the most critical coordinating histidine residue (H55) to screen for activity with histidine-like ncAAs, identifying πMH as the best and only candidate screened with sufficient activity at slightly acidic pH. Characterization of the H55πMH substitution revealed that the variant could be efficiently expressed either as a dizinc or dicobalt enzyme. Previous studies with πMH mutagenesis of zinc-coordinating histidine residues have been conducted to create ncAA-dependent cells^59^ and to study an allosteric regulatory mechanism governed by switching between πN/τN histidine coordination.^60^ In both of these enzyme systems, the substitution negatively impacted function. In contrast, we found that the H55πMH variant of dPTE2 not only retained activity but exhibited elevated activity at low pH, with improvements up to 5-fold that of the parent enzyme.

Mechanistic and structure–function conclusions regarding the effect of the H55πMH substitution were hampered by challenges related to fully assessing the activity of high p*K*_a_^LG^ substrates and structure at low pH. Thus, the molecular basis for the improved activity with H55πMH at low pH remains to be fully elucidated. Nonetheless, our mechanistic analysis indicates that the H55πMH substitution corresponds to a reduction in p*K*_a_^cat^, an increase in the catalytic competence of the acidic pathway, and an increase in *k*_5_, corresponding to irreversible steps following the P–O bond cleavage. Structurally, the methyl group of πMH displaces an ordered water molecule observed in the H55 active site, potentially altering the electrostatic environment or hydrogen bonding network. Additionally, although πMH and histidine have similar conjugate acid p*K*_a_ values (6.7 vs 6.5), subtle differences in σ-donation or metal–ligand bond polarizability could influence the p*K*_a_ of the bridging hydroxide or affect the binding affinity of the aspartate and carboxylated lysine ligands. Previous work with heme and nonheme enzymes indicates that the πMH can be less electron donating as an iron ligand when it is polarized through hydrogen bonding to a carboxylate side chain form the imidazolate-iron complex.^26,61^ If the water-H55 interaction is acting similarly, then the H55πMH substitution may provide a weaker donor, creating a stronger metal-hydroxide bond, lowering the catalytic p*K*_a_, and forming a more reactive ESH+ complex. Additionally, changes in steps following P–O bond cleavage, reflected in the increased *k*_5_ values, may arise from alteration in the interaction with the phosphate product, altered kinetics for reconstruction of the active free enzyme, or modification of commitment factors related to a pre-equilibrium before *k*_5_. Upon comparing the zinc and cobalt systems, cobalt appears to diminish *k*_2_ and increase *k*_5_. The changes correlate well with the observed *k*_5_ differences between zinc and cobalt, suggesting that these alterations are the source of benefit with the “slow” substrates. Evaluating these hypotheses remains a goal for future studies.

In summary, we find that application of genetic code expansion to modify this critical metal-coordinating histidine enabled more nuanced study of the role of this residue and enabled engineering to produce an enzyme variant with up to 5-fold higher catalytic activity at low pH. These significant advances add to the growing support for ncAA engineering as a valuable tool for studying and improving protein function^22,27–29,58,62–69^ and highlight opportunities for probing diverse metal coordination sites.

## Supplementary Material



## Notes

Activity data of the ncAA panel screened at position H55; LC-MS and MS/MS data; metal quantification results; calibration curves determined from the absorbance of the phenol products from 0–1000 μM; Michaelis–Menten curves for pH profiles of substrates I–V; comparison of model fits for log­(*k*_cat_, s^–1^); Michaelis–Menten curves for pD profiles of substrates **III** and **IV**; pD vs pH analysis; proposed mechanistic model; Brønsted plots for pH 7.5 and 8.0; tables for kinetic and structural data; chemical synthesis information; NMR data; and DNA and protein sequences (PDF)
