# Supplementary material for: Catalytic pK a Attenuation in a Hydrolytic Metalloenzyme by Genetic Code Expansion
Source: Biochemistry. 2026 Feb 18;65(5):559–70. doi: 10.1021/acs.biochem.5c00768 (PMC12961738; doi:10.1021/acs.biochem.5c00768)
Supplement: Supplementary file 1 [file bi5c00768_si_001.pdf]

Supporting information: Catalytic pK_a Attenuation in a Hydrolytic Metalloenzyme by Genetic Code Expansion

Benjamin P. Manser[1], Alexandria Deliz Liang[1]*

[1]Department of Chemistry, University of Zurich; Winterthurerstrasse 190, 8057, Zurich, Switzerland.

*Correspondence should be addressed to A.D.L. (alexandriadeliz.liang@uzh.ch).

Table of Contents

I.	Supplementary data figures	S2
II.	Supplementary data tables	S18
III.	Chemical synthesis	S28
IV.	NMR spectra	S30
V.	DNA and Protein Sequences	S34
VI.	References	S37

I. Supplementary data figures

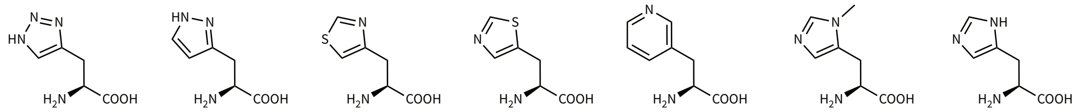

ncAA	123Trz-4A	3PzA	4ThzA	5ThzA	3PyA	πMH	His
Zn/monomer	2.2	2.4	1.7	0.65	2.1	2.0	2.0
k_{cat} (s^{-1})	49 (36-64)	n.d.	n.d.	110 (100-120)	100 (78-130)	970 (880-1100)	1000 (920-1100)
k_{cat}/K_m ($s^{-1}\mu M^{-1}$)	2.3 (0.61-12)	n.d.	n.d.	2.1 (1.6-2.9)	0.43 (0.22-0.88)	20 (14-28)	23 (16-32)

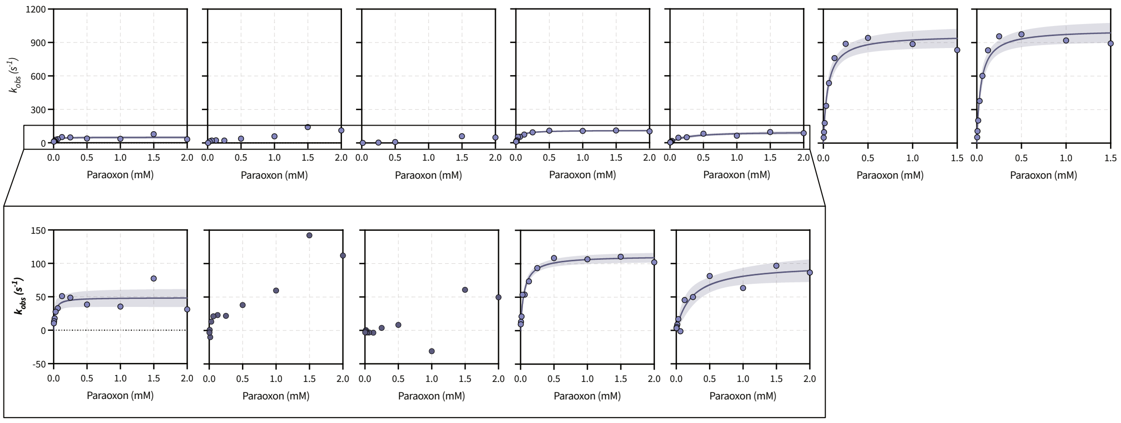

Supplementary Figure 1. Activity data of the histidine analogue panel screened at position H55(ncAA) in the full strepTagII-SUMO-(tev)-dPTE2 construct for substrate III at pH 7. The data represent a single biological replicate. Errors for the extracted kinetic constants are given in parentheses as 95% CI lower and upper bounds. Only the πMH variant retains significant activity compared to the parent His variant. For 5ThzA, the loss of activity may result from impaired metal binding.

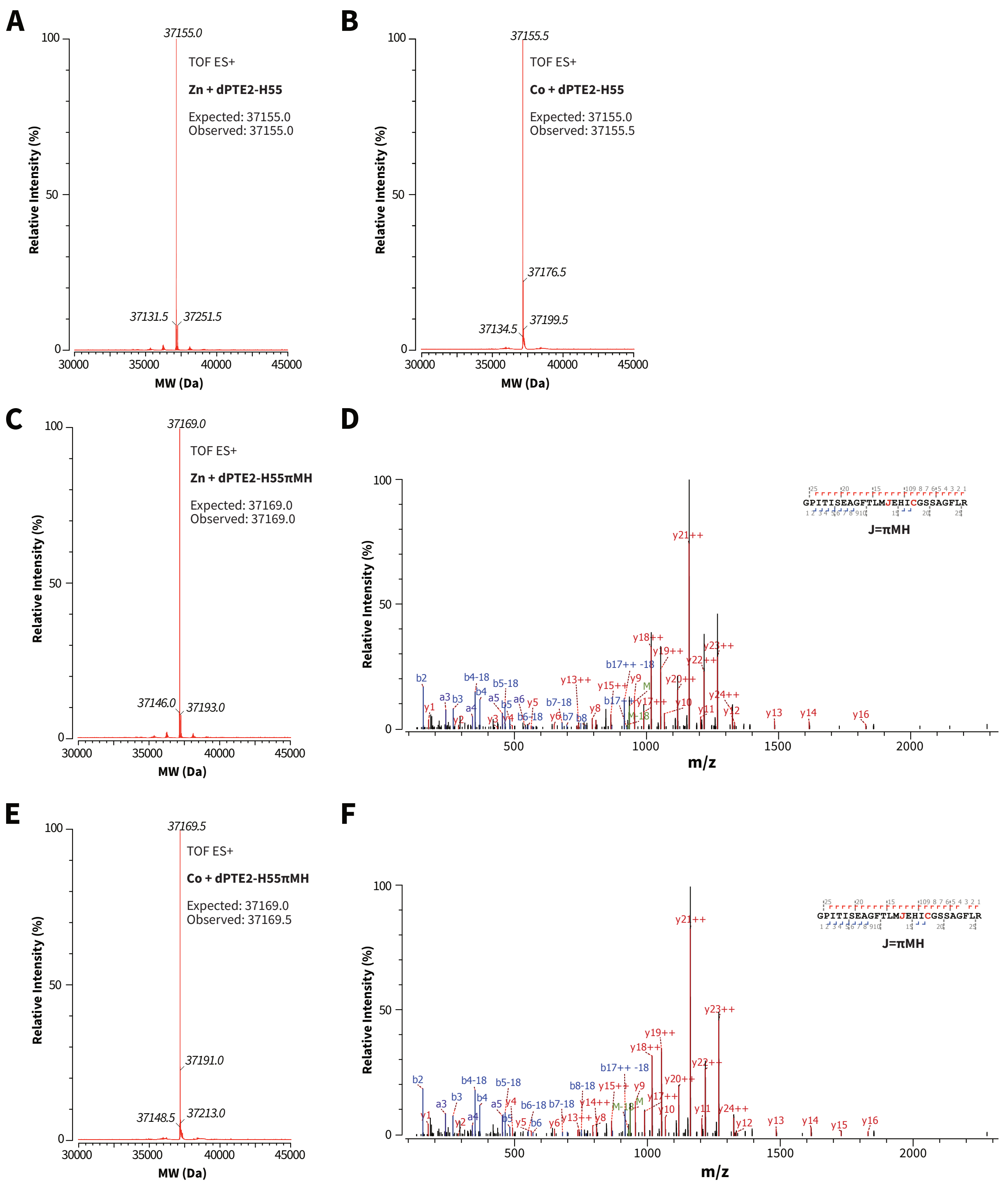

Supplementary Figure 2. LC-MS and LC-MS/MS data of the trypsinized protein, confirming protein identity and incorporation of πMH.

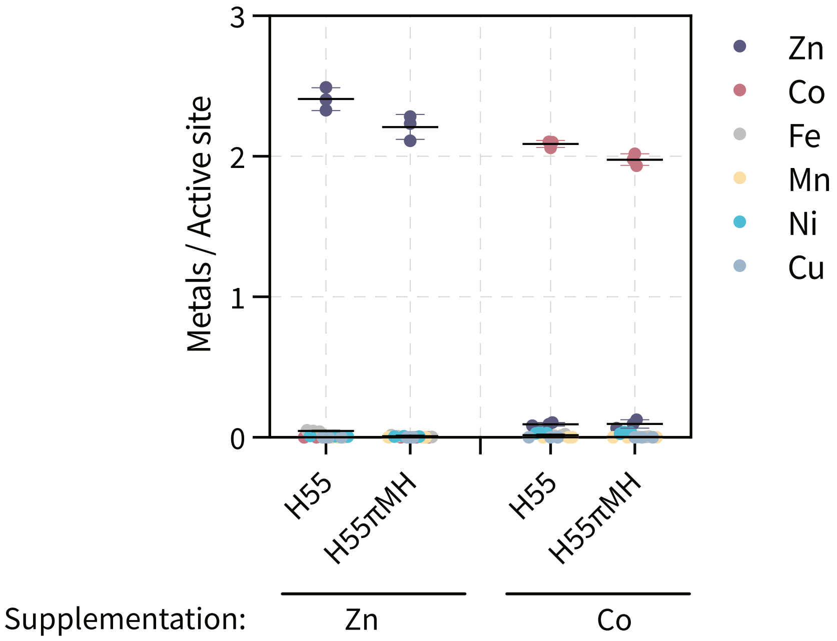

Supplementary Figure 3. ICP-MS data confirming correct metal substitution. Concentration of several metals known to be capable of occupying the PTE active site were assessed. Only the desired zinc and cobalt were detected in significant concentrations.

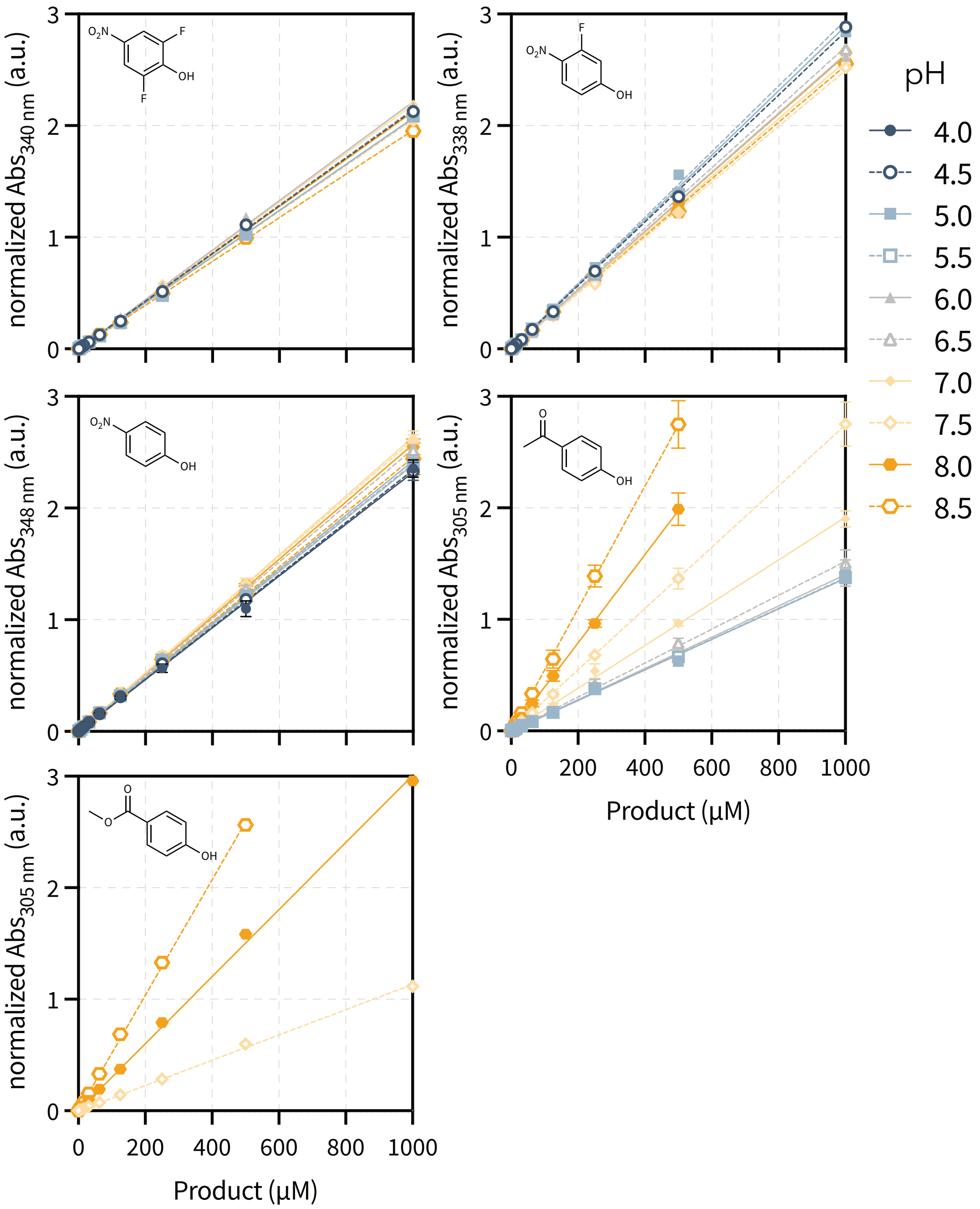

Supplementary Figure 4. Calibration curves determined from the absorbance of the phenol products from 0 - 1000 µM.

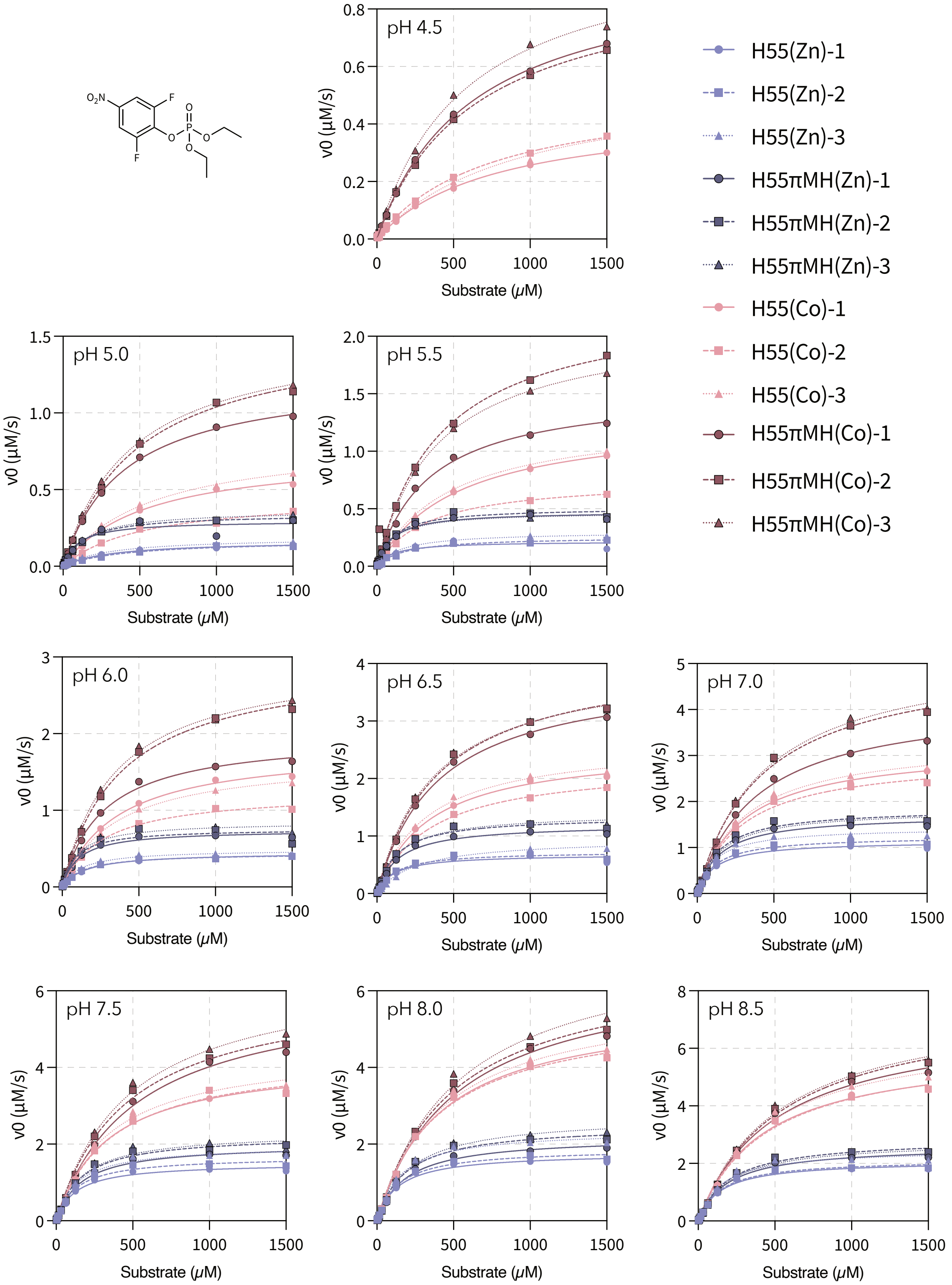

Supplementary Figure 5. Individual Michaelis-Menten data for the hydrolysis of substrate I at varying pH. Data points represent a single measurement. The line represents the fit to the Michaelis-Menten equation: $v = \frac{V_{max}*A}{(K_m+A)}$

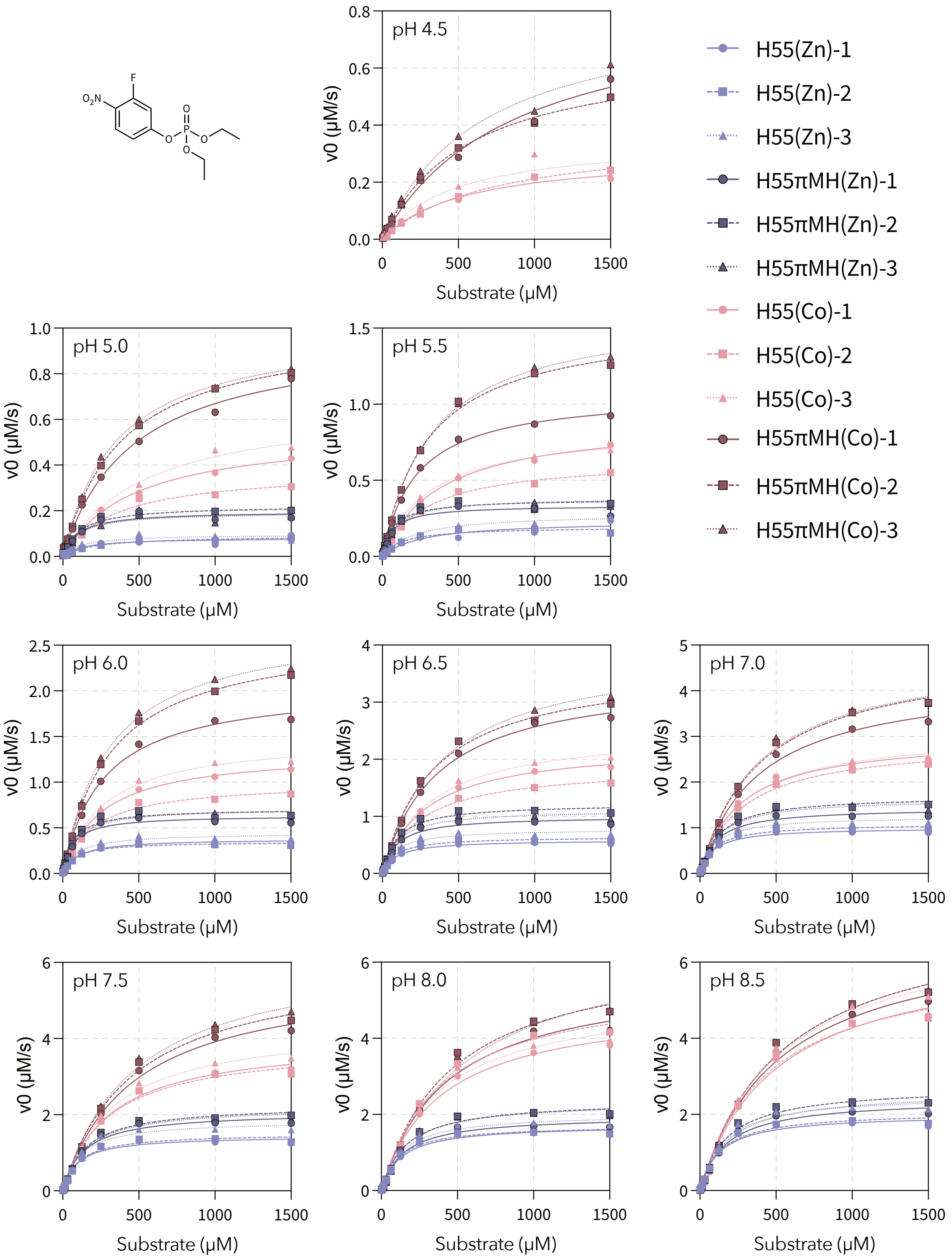

Supplementary Figure 6. Individual Michaelis-Menten data for the hydrolysis of substrate **II** at varying pH. Data points represent a single measurement. The line represents the fit to the Michaelis-Menten equation: $v = \frac{V_{max} * A}{(K_m + A)}$

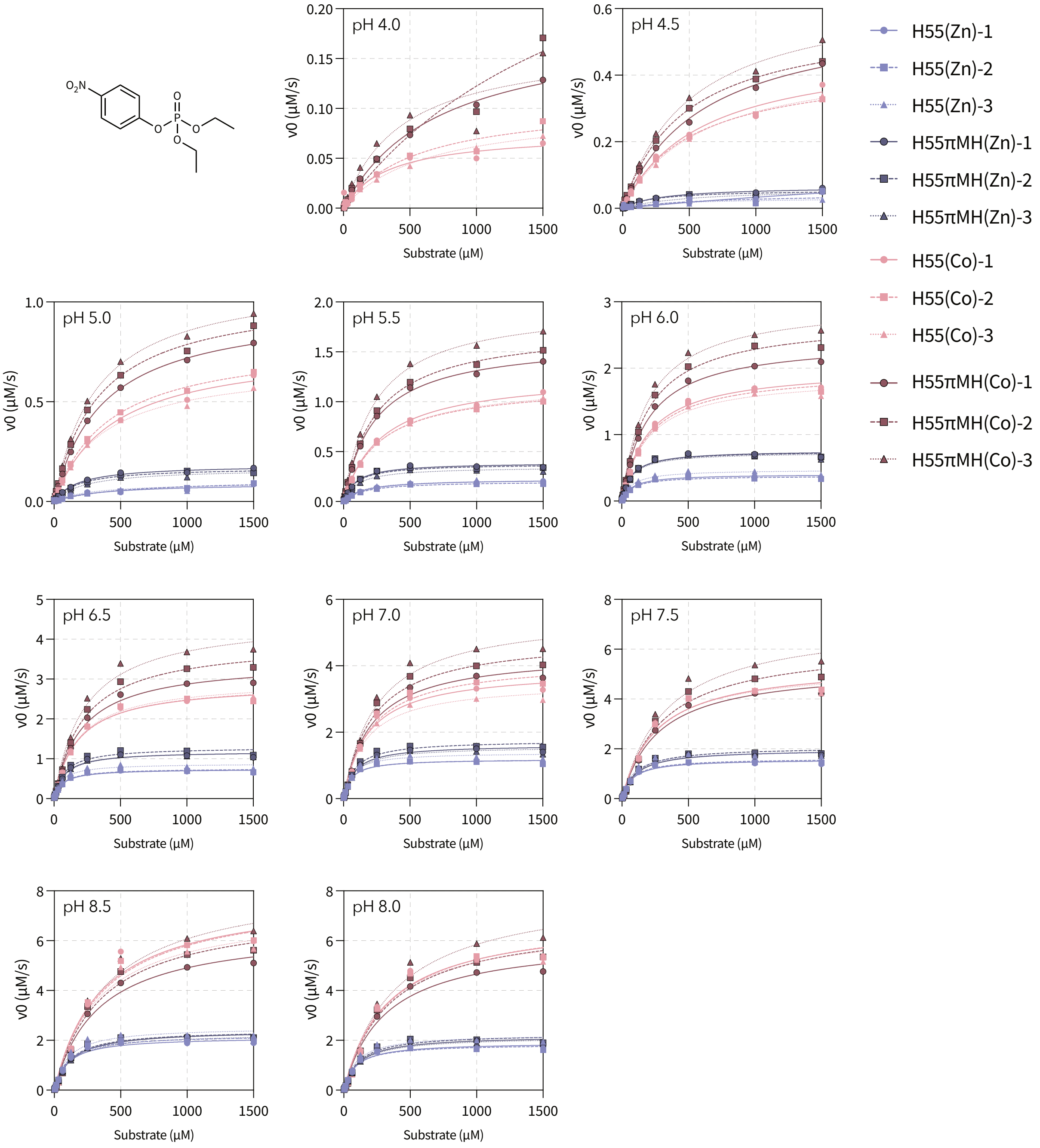

Supplementary Figure 7. Individual Michaelis-Menten data for the hydrolysis of substrate **III** at varying pH. Data points represent a single measurement. The line represents the fit to the Michaelis-Menten equation: $v = \frac{V_{max}*A}{(K_m+A)}$

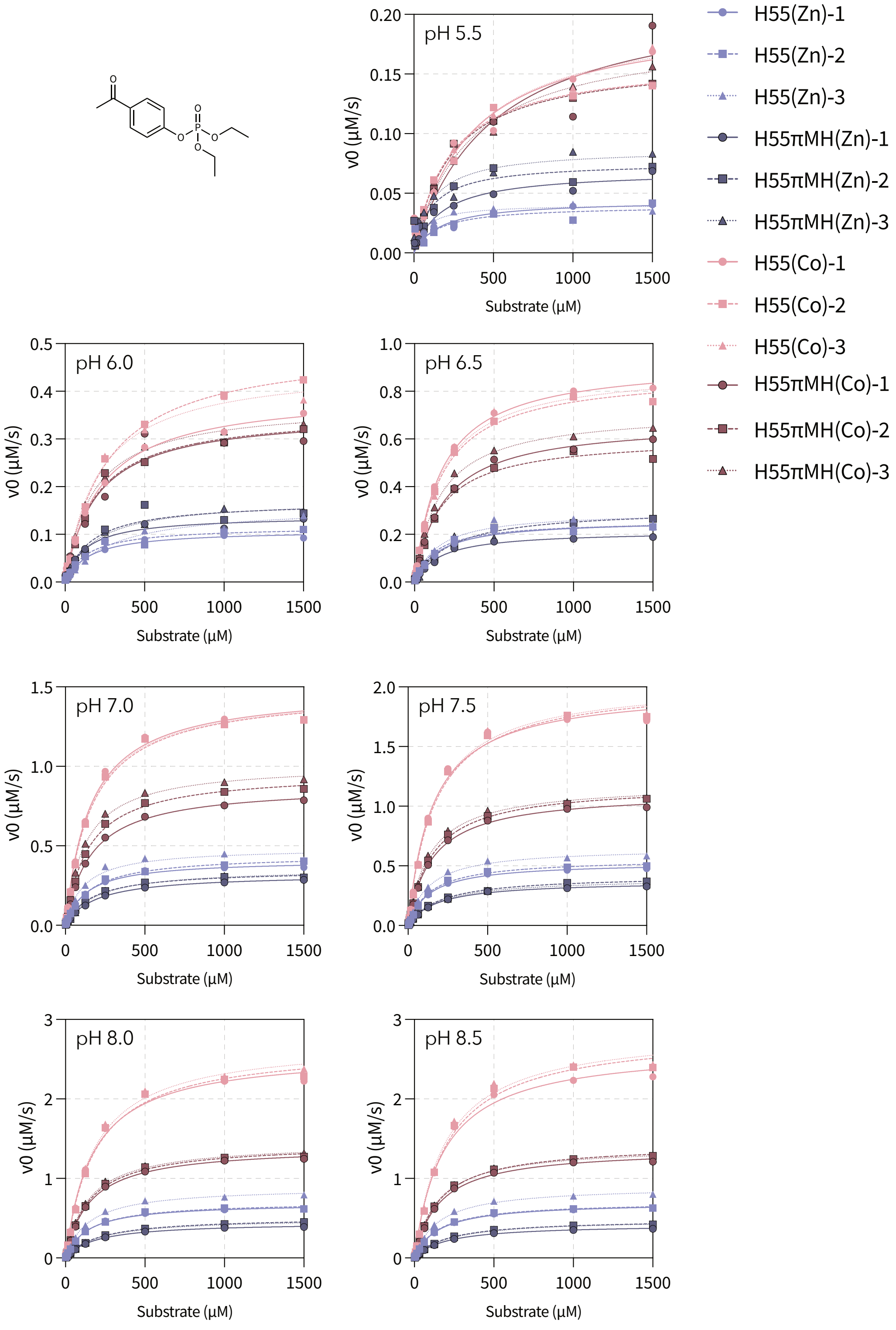

Supplementary Figure 8. Individual Michaelis-Menten data for the hydrolysis of substrate IV at varying pH. Data points represent a single measurement. The line represents the fit to the Michaelis-Menten equation: $v = \frac{V_{max}*A}{(K_m+A)}$

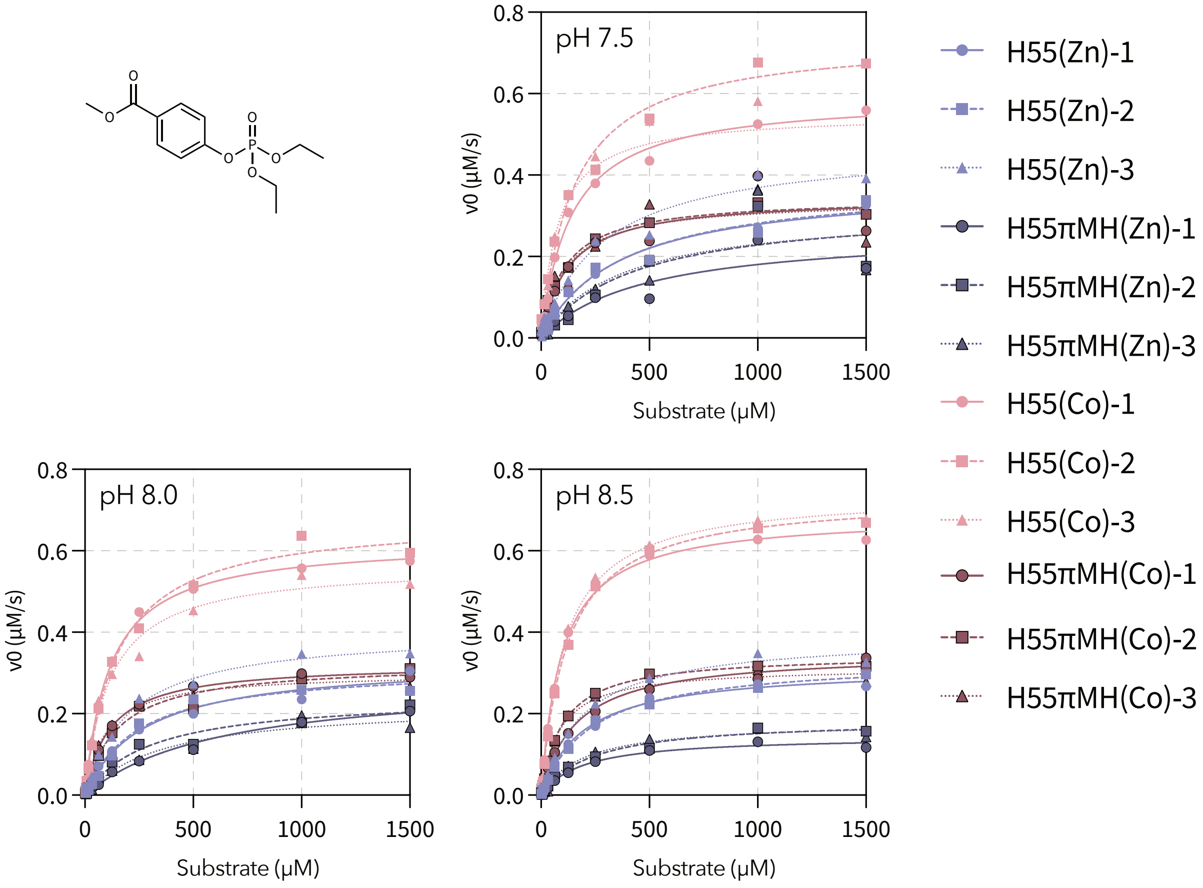

Supplementary Figure 9. Individual Michaelis-Menten data for the hydrolysis of substrate V at varying pH. Data points represent a single measurement. The line represents the fit to the Michaelis-Menten equation: $v = \frac{V_{max}*A}{(K_m+A)}$

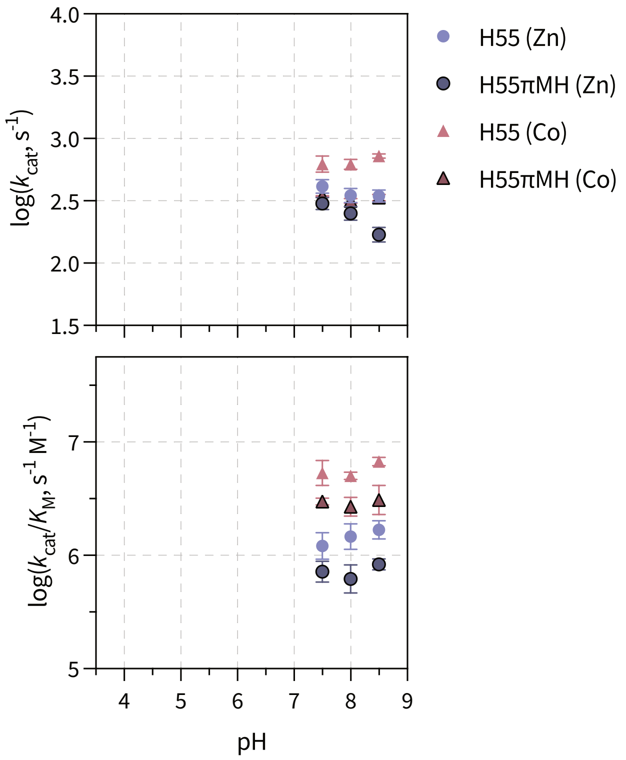

Supplementary Figure 10. Activity data at pH 7.5, 8.0, and 8.5 for substrate V. The activity below 7.5 could not be reliably assessed.

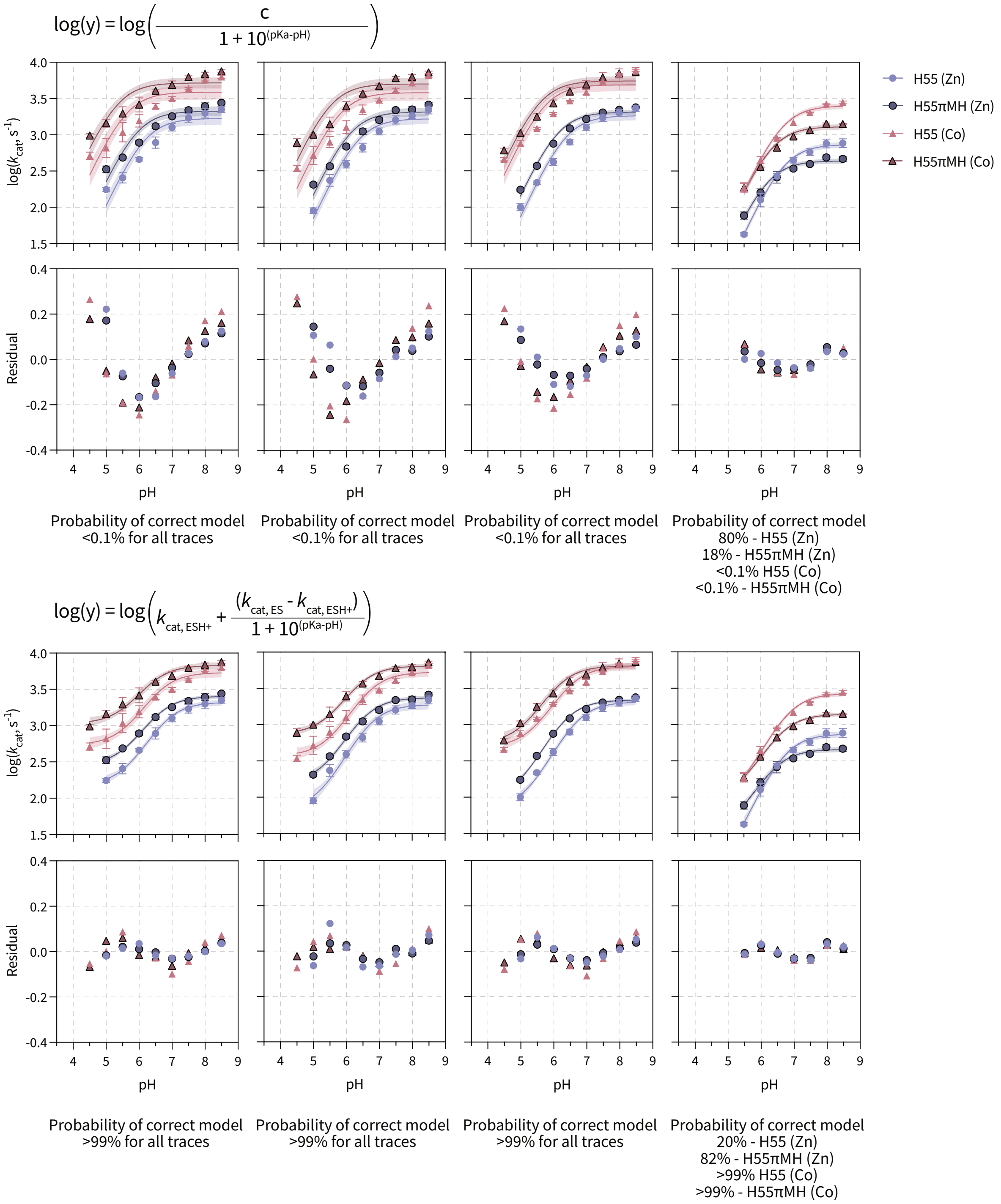

Supplementary Figure 11. Model comparison for fitting the k_{cat} data. The overlayed fits (agreement between predicted and experimental), the residuals (shape, distribution, and homoscedasticity), and the AIC results (model probabilities) all support the second, more complex model.

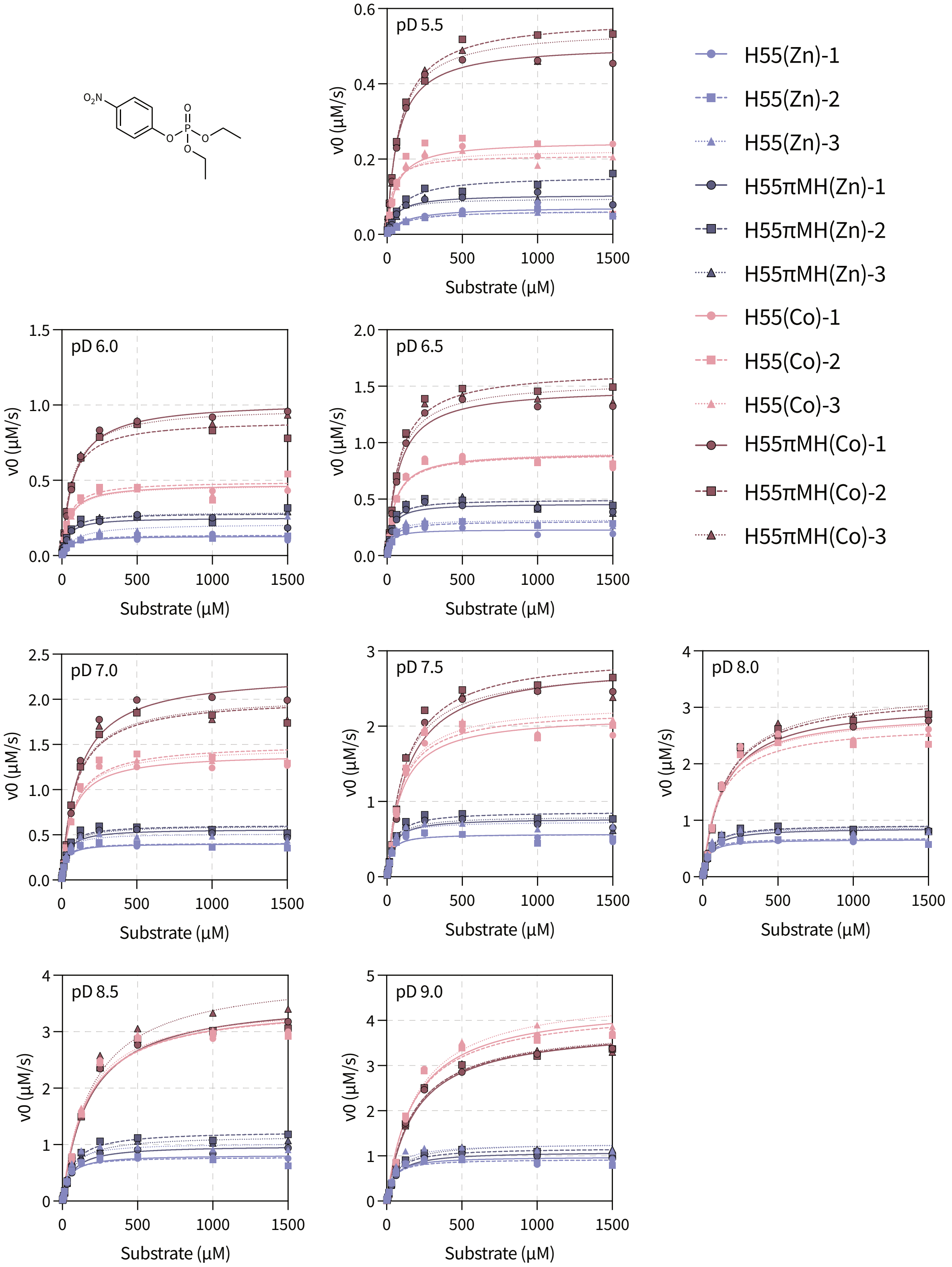

Supplementary Figure 12. Michaelis-Menten curves for D_2O experiments with substrate III.

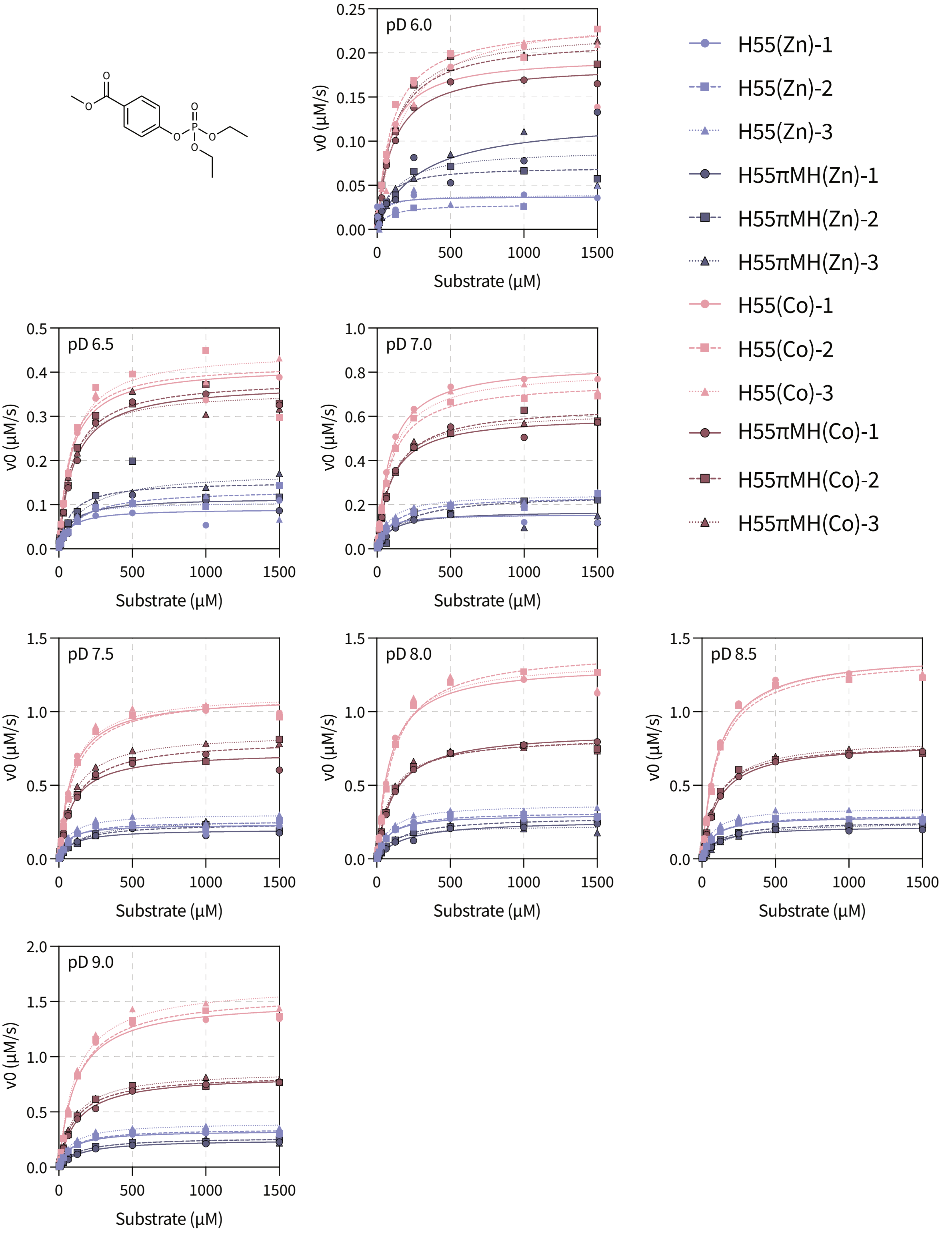

Supplementary Figure 13. Michaelis-Menten curves for D_2O experiments with substrate IV.

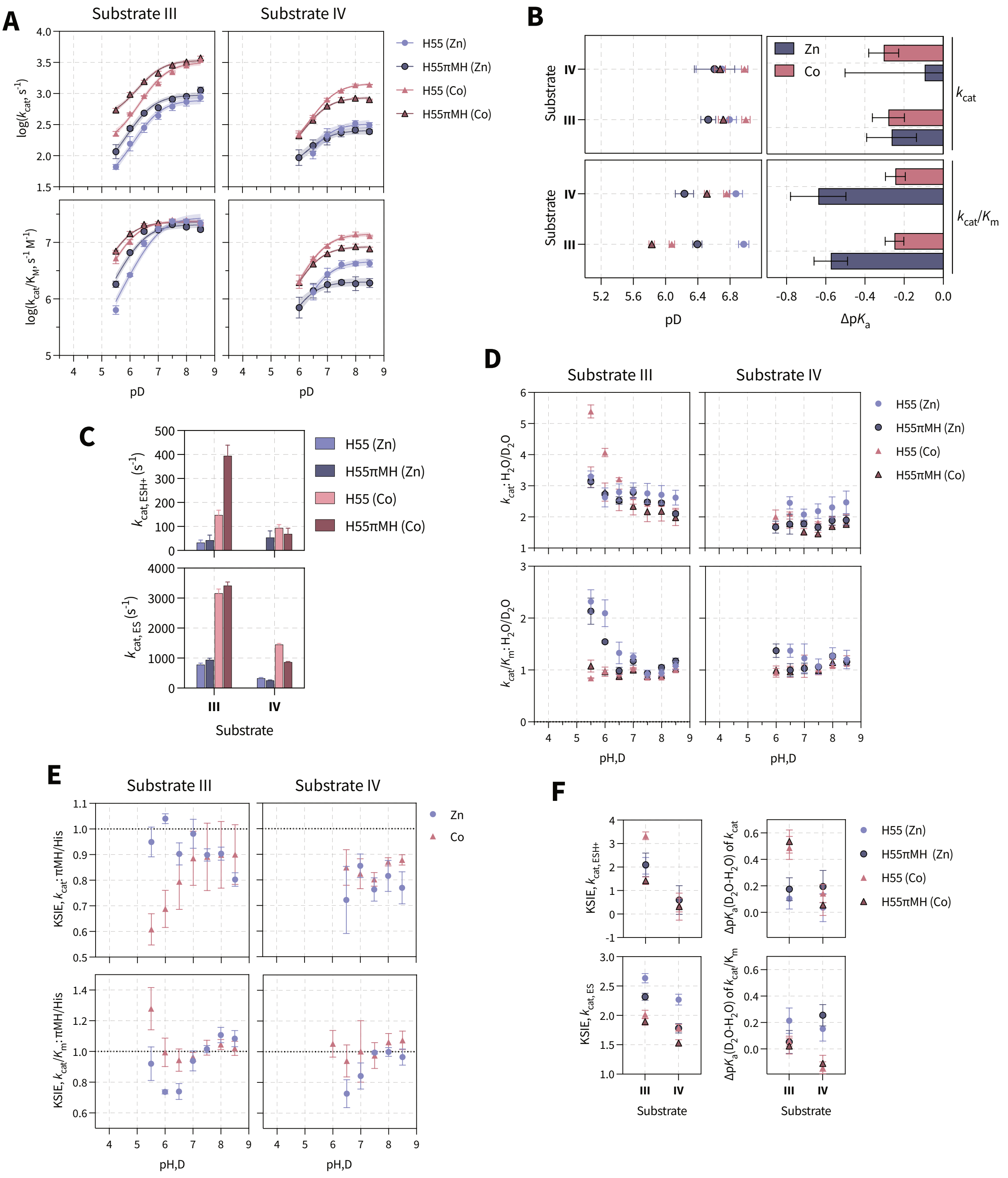

Supplementary Figure 14. Deuterium solvent isotope effect studies in >95% D_2O for substrates **III** and **IV**. These substrates were selected because they well represent the two different classes of substrates—those "slow substrates" (high pK_a^{LG}) and "fast substrates" (low pK_a^{LG}). A) pH-rate profiles. Data points represent the mean of three biological replicates; the error bars represent standard deviation; and the shading indicates the 95% CI of the pK_a fit to the equation: $log(y) = log\left(k_{\text{cat,ESH+}} + \frac{(k_{\text{cat,ES}} - k_{\text{cat,ESH+}})}{(1+10^{(pKa-\text{pH})})}\right)$ or $log(y) = log\left(\frac{c}{(1+10^{(pKa-\text{pH})})}\right)$ .B) From the pH profile analysis, the observed catalytic pK_as were extracted and the changes upon substituting histidine with πMH were determined. The bars represent the mean of three biological replicates, and the error bars represent the standard deviation. C) For the k_{cat} model, the rate constants for the lower activity protonated state (top, ESH+) and for the higher activity deprotonated state (bottom, ES)

were extracted. D) Kinetic solvent isotope effects (KSIEs). E) πMH/His ratios of the KSIEs. F) KSIEs of $k_{cat, ESH+}$ and $k_{cat, ES}$.

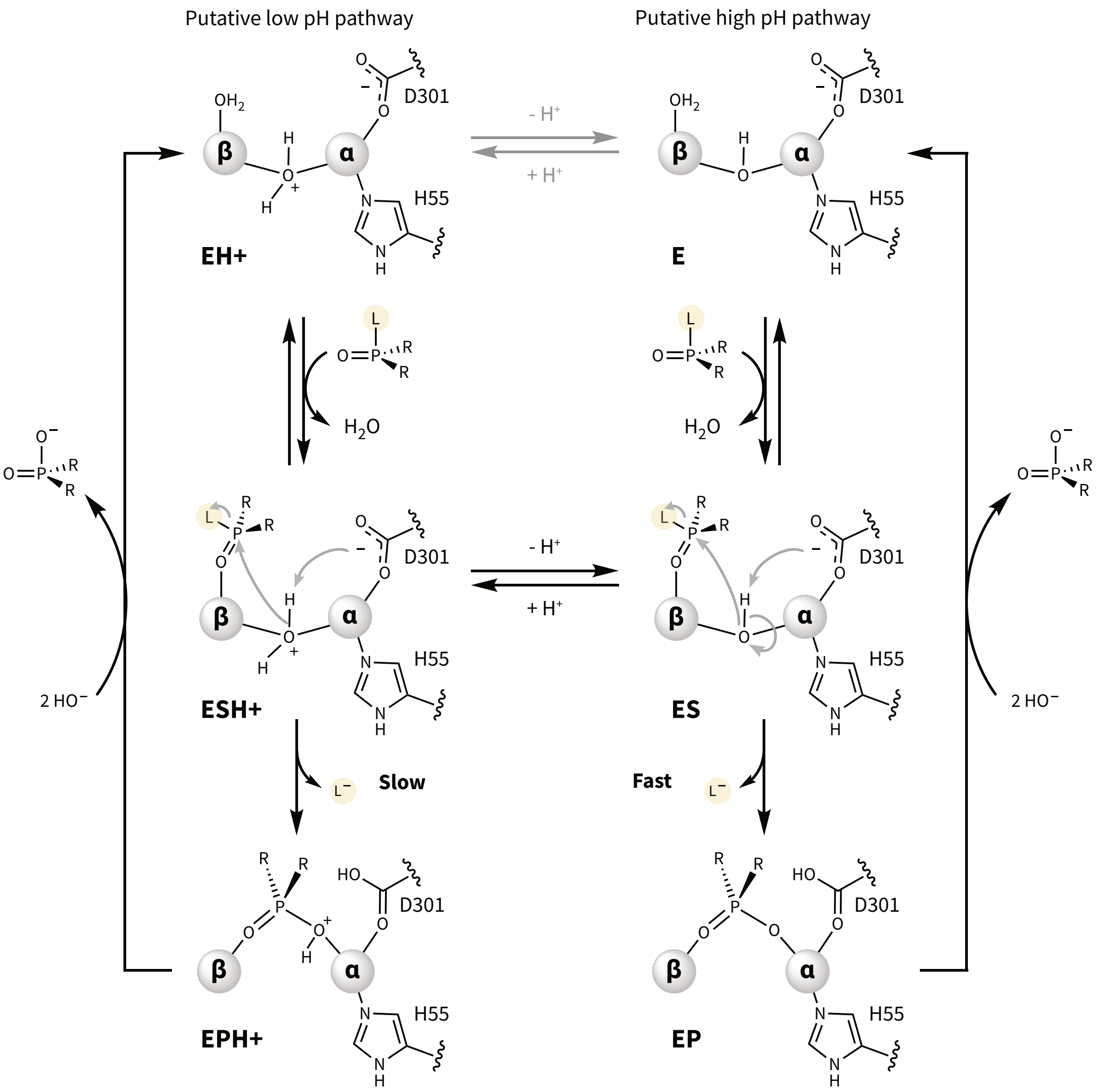

Supplementary Figure 15. A proposed model for the two ES complexes in which the single ionizable species observed in the k_{cat} is the bridging aquo/hydroxo ligand. The model assumes a rapid equilibrium between E and EH+.

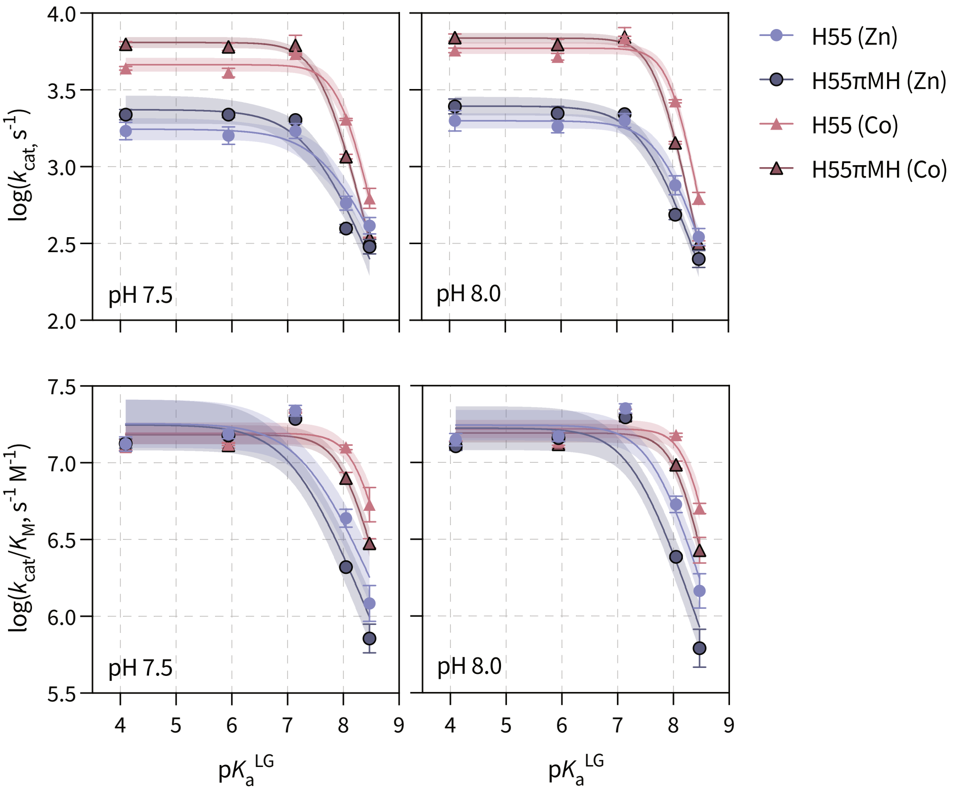

Supplementary Figure 16. Brønsted plot for the dependence of log(k_{cat}) and log(k_{cat}/K_m) on the pK_a^{LG} at pH 8.0 and 7.5. Data points represent the mean of three biological replicates; the error bars represent standard deviation; and the shading indicates the 95% CI of the model fit to the equations: $log(k_{cat}) = log\left(\frac{k_3 k_5}{k_3+k_5}\right)$ or $log\left(\frac{k_{cat}}{K_M}\right) = log\left(\frac{k_1 k_3}{k_2+k_3}\right)$, with the Brønsted catalysis equation $\log(k_3) = \beta pK_a^{LG} + C$.

II. Supplementary data tables

Supplementary Table 1. log(k_{cat}, s^{-1}) values for substrate I. Mean of three biological replicates. Error values as standard deviation

pH	Variant			
	H55(Zn)	H55πMH(Zn)	H55(Co)	H55πMH(Co)
8.5	3.35±0.04	3.44±0.02	3.80±0.02	3.88±0.02
8.0	3.30±0.07	3.39±0.05	3.76±0.02	3.84±0.02
7.5	3.23±0.06	3.34±0.03	3.64±0.01	3.80±0.02
7.0	3.10±0.05	3.25±0.02	3.50±0.03	3.69±0.05
6.5	2.89±0.08	3.12±0.03	3.40±0.04	3.61±0.01
6.0	2.66±0.02	2.89±0.03	3.19±0.09	3.42±0.10
5.5	2.41±0.07	2.69±0.02	3.04±0.13	3.29±0.09
5.0	2.24±0.03	2.52±0.05	2.82±0.13	3.16±0.05
4.5	-	-	2.71±0.05	2.99±0.03

Supplementary Table 2. log(k_{cat}, s^{-1}) values for substrate II. Mean of three biological replicates. Error values as standard deviation

pH	Variant			
	H55(Zn)	H55πMH(Zn)	H55(Co)	H55πMH(Co)
8.5	3.34±0.05	3.41±0.03	3.82±0.03	3.86±0.02
8.0	3.26±0.04	3.35±0.05	3.72±0.02	3.80±0.03
7.5	3.20±0.06	3.34±0.02	3.61±0.03	3.78±0.02
7.0	3.05±0.05	3.20±0.04	3.48±0.01	3.67±0.03
6.5	2.82±0.06	3.05±0.04	3.34±0.07	3.57±0.03
6.0	2.59±0.05	2.84±0.03	3.10±0.09	3.39±0.07
5.5	2.37±0.08	2.56±0.03	2.90±0.08	3.15±0.10
5.0	1.95±0.04	2.31±0.03	2.72±0.12	3.00±0.01
4.5	-	-	2.53±0.04	2.89±0.06

Supplementary Table 3. log(k_{cat}, s^{-1}) values for substrate III. Mean of three biological replicates. Error values as standard deviation

pH	Variant			
	H55(Zn)	H55πMH(Zn)	H55(Co)	H55πMH(Co)
8.5	3.36±0.04	3.38±0.01	3.89±0.02	3.87±0.06
8.0	3.30±0.05	3.34±0.01	3.84±0.01	3.84±0.06
7.5	3.23±0.05	3.30±0.01	3.73±0.01	3.79±0.06
7.0	3.10±0.04	3.22±0.02	3.59±0.04	3.69±0.05
6.5	2.90±0.04	3.09±0.02	3.47±0.01	3.60±0.06
6.0	2.62±0.05	2.88±0.01	3.29±0.01	3.43±0.05
5.5	2.34±0.02	2.57±0.03	3.09±0.02	3.25±0.04
5.0	2.00±0.05	2.24±0.02	2.88±0.04	3.02±0.03
4.5	-	-	2.66±0.02	2.78±0.03

Supplementary Table 4. log(k_{cat}, s^{-1}) values for substrate IV. Mean of three biological replicates. Error values as standard deviation

pH	Variant			
	H55(Zn)	H55πMH(Zn)	H55(Co)	H55πMH(Co)
8.5	2.88±0.06	2.67±0.04	3.45±0.02	3.15±0.01
8.0	2.88±0.06	2.69±0.03	3.43±0.01	3.16±0.01
7.5	2.76±0.05	2.60±0.03	3.31±0.01	3.07±0.01
7.0	2.65±0.04	2.53±0.02	3.17±0.00	2.98±0.03
6.5	2.43±0.03	2.41±0.08	2.95±0.01	2.82±0.04
6.0	2.10±0.09	2.20±0.05	2.65±0.05	2.56±0.01
5.5	1.63±0.02	1.89±0.05	2.27±0.05	2.27±0.07
5.0	-	-	-	-

Supplementary Table 5. log(k_{cat}, s^{-1}) values for substrate V. Mean of three biological replicates. Error values as standard deviation

pH	Variant			
	H55(Zn)	H55πMH(Zn)	H55(Co)	H55πMH(Co)
8.5	2.54±0.05	2.23±0.06	2.86±0.02	2.53±0.03
8.0	2.54±0.06	2.40±0.05	2.79±0.04	2.50±0.02
7.5	2.62±0.05	2.48±0.05	2.79±0.07	2.54±0.00

Supplementary Table 6. log(k_{cat}/K_m, $s^{-1}M^{-1}$) values for substrate I. Mean of three biological replicates. Error values as standard deviation

pH	Variant			
	H55(Zn)	H55πMH(Zn)	H55(Co)	H55πMH(Co)
8.5	7.19±0.04	7.15±0.01	7.16±0.02	7.15±0.01
8.0	7.15±0.04	7.11±0.03	7.14±0.00	7.13±0.01
7.5	7.12±0.05	7.13±0.03	7.10±0.02	7.11±0.04
7.0	7.04±0.06	7.09±0.03	7.03±0.01	7.07±0.03
6.5	6.73±0.13	6.99±0.04	6.87±0.05	7.02±0.02
6.0	6.52±0.07	6.91±0.06	6.69±0.03	6.90±0.03
5.5	6.24±0.09	6.69±0.01	6.40±0.04	6.71±0.05
5.0	5.71±0.09	6.43±0.05	6.09±0.12	6.51±0.02
4.5	-	-	5.80±0.05	6.20±0.05

Supplementary Table 7. log(k_{cat}/K_m, $s^{-1}M^{-1}$) values for substrate II. Mean of three biological replicates. Error values as standard deviation

pH	Variant			
	H55(Zn)	H55πMH(Zn)	H55(Co)	H55πMH(Co)
8.5	7.20±0.02	7.17±0.02	7.13±0.02	7.13±0.00
8.0	7.17±0.03	7.16±0.02	7.14±0.02	7.12±0.03
7.5	7.19±0.03	7.18±0.01	7.12±0.01	7.11±0.02
7.0	7.09±0.04	7.13±0.00	7.04±0.03	7.08±0.03
6.5	6.88±0.08	7.07±0.03	6.89±0.01	7.01±0.02
6.0	6.62±0.05	6.98±0.03	6.73±0.02	6.92±0.03
5.5	6.08±0.12	6.68±0.05	6.39±0.05	6.69±0.02
5.0	5.78±0.14	6.29±0.02	6.07±0.03	6.40±0.07
4.5	-	-	5.78±0.09	6.06±0.06

Supplementary Table 8. log(k_{cat}/K_m, $s^{-1}M^{-1}$) values for substrate III. Mean of three biological replicates. Error values as standard deviation

pH	Variant			
	H55(Zn)	H55πMH(Zn)	H55(Co)	H55πMH(Co)
8.5	7.37±0.03	7.30±0.02	7.35±0.01	7.32±0.02
8.0	7.35±0.03	7.29±0.02	7.33±0.02	7.31±0.01
7.5	7.34±0.04	7.29±0.01	7.33±0.01	7.31±0.02
7.0	7.33±0.03	7.29±0.03	7.33±0.01	7.34±0.01
6.5	7.10±0.07	7.19±0.03	7.21±0.00	7.26±0.03
6.0	6.74±0.05	7.01±0.01	7.00±0.01	7.14±0.04
5.5	6.17±0.04	6.59±0.05	6.65±0.01	6.88±0.05
5.0	5.43±0.09	5.95±0.08	6.27±0.02	6.49±0.06
4.5	-	5.27±0.18	5.91±0.02	6.07±0.06
4.0	-	-	5.28±0.08	5.42±0.16

Supplementary Table 9. log(k_{cat}/K_m, $s^{-1}M^{-1}$) values for substrate IV. Mean of three biological replicates. Error values as standard deviation

pH	Variant			
	H55(Zn)	H55πMH(Zn)	H55(Co)	H55πMH(Co)
8.5	6.71±0.06	6.35±0.02	7.16±0.01	6.97±0.02
8.0	6.73±0.05	6.39±0.01	7.18±0.01	6.99±0.02
7.5	6.64±0.06	6.32±0.00	7.10±0.02	6.90±0.04
7.0	6.53±0.09	6.28±0.04	6.97±0.01	6.83±0.09
6.5	6.27±0.04	6.16±0.05	6.74±0.01	6.60±0.05
6.0	5.84±0.10	6.01±0.04	6.29±0.04	6.29±0.04
5.5	5.60±0.25	5.77±0.16	5.79±0.07	5.75±0.10
5.0	-	-	-	-

Supplementary Table 10. log(k_{cat}/K_m, $s^{-1}M^{-1}$)values for substrate V. Mean of three biological replicates. Error values as standard deviation

pH	Variant			
	H55(Zn)	H55πMH(Zn)	H55(Co)	H55πMH(Co)
8.5	6.22±0.08	5.92±0.05	6.83±0.04	6.49±0.13
8.0	6.16±0.11	5.79±0.12	6.70±0.03	6.43±0.08
7.5	6.08±0.12	5.86±0.09	6.73±0.11	6.48±0.03

Supplementary Table 11. log(k_{cat}, s^{-1}) values for substrate III in >95% D_2O. Mean of three biological replicates. Error values as standard deviation

pH	Variant			
	H55(Zn)	H55πMH(Zn)	H55(Co)	H55πMH(Co)
8.5	2.94±0.06	3.05±0.05	3.55±0.00	3.57±0.03
8.0	2.87±0.07	2.95±0.02	3.45±0.02	3.51±0.02
7.5	2.79±0.06	2.91±0.03	3.35±0.01	3.46±0.01
7.0	2.64±0.06	2.77±0.02	3.17±0.02	3.33±0.03
6.5	2.45±0.08	2.68±0.02	2.96±0.00	3.19±0.02
6.0	2.19±0.12	2.44±0.03	2.68±0.01	2.99±0.03
5.5	1.82±0.04	2.07±0.11	2.35±0.04	2.73±0.03

Supplementary Table 12. log(k_{cat}, s^{-1}) values for substrate IV in >95% D_2O. Mean of three biological replicates. Error values as standard deviation

pH	Variant			
	H55(Zn)	H55πMH(Zn)	H55(Co)	H55πMH(Co)
8.5	2.49±0.04	2.39±0.04	3.14±0.00	2.90±0.01
8.0	2.52±0.05	2.41±0.05	3.14±0.02	2.93±0.01
7.5	2.42±0.06	2.37±0.07	3.05±0.00	2.90±0.04
7.0	2.32±0.11	2.28±0.10	2.91±0.02	2.80±0.02
6.5	2.04±0.09	2.16±0.09	2.63±0.02	2.57±0.02
6.0	-	1.97±0.13	2.35±0.04	2.32±0.04

Supplementary Table 13. log(k_{cat}/K_m, $s^{-1}M^{-1}$) values for substrate III in >95% D_2O. Mean of three biological replicates. Error values as standard deviation

pH	Variant			
	H55(Zn)	H55πMH(Zn)	H55(Co)	H55πMH(Co)
8.5	7.34±0.06	7.23±0.03	7.35±0.01	7.31±0.00
8.0	7.38±0.04	7.27±0.03	7.40±0.00	7.35±0.00
7.5	7.38±0.02	7.31±0.01	7.40±0.02	7.37±0.04
7.0	7.24±0.05	7.22±0.03	7.31±0.00	7.34±0.02
6.5	6.98±0.06	7.20±0.03	7.24±0.01	7.32±0.02
6.0	6.42±0.03	6.82±0.02	7.01±0.04	7.16±0.04
5.5	5.80±0.08	6.26±0.06	6.72±0.10	6.85±0.02

Supplementary Table 14. log(k_{cat}/K_m, $s^{-1}M^{-1}$) values for substrate IV in >95% D_2O. Mean of three biological replicates. Error values as standard deviation

pH	Variant			
	H55(Zn)	H55πMH(Zn)	H55(Co)	H55πMH(Co)
8.5	6.63±0.06	6.28±0.08	7.11±0.02	6.89±0.03
8.0	6.62±0.04	6.27±0.12	7.14±0.04	6.93±0.05
7.5	6.61±0.07	6.29±0.04	7.09±0.04	6.90±0.04
7.0	6.44±0.10	6.25±0.13	6.94±0.03	6.80±0.02
6.5	6.12±0.14	6.15±0.13	6.72±0.02	6.62±0.04
6.0	-	5.85±0.18	6.31±0.12	6.29±0.03

Supplementary Table 15. pK_a^{cat} values for k_{cat} from a fit of the pH-activity data with Eq.2. Mean of three biological replicates. Error values as SEM

M^{2+}	Position 55	Substrate			
		I	II	III	IV
Zn	His	6.79±0.06	6.69±0.08	6.69±0.05	6.74±0.06
	πMH	6.57±0.05	6.54±0.05	6.35±0.03	6.41±0.08
Co	His	6.62±0.10	6.68±0.08	6.51±0.08	6.75±0.05
	πMH	6.40±0.09	6.41±0.06	6.84±0.04	6.62±0.05

Supplementary Table 16. pK_a^{cat} values for k_{cat}/K_m from a fit of the pH-activity data with Eq.1. Mean of three biological replicates. Errors values as SEM

M^{2+}	Position 55	Substrate			
		I	II	III	IV
Zn	His	6.44±0.05	6.49±0.04	6.76±0.07	6.72±0.07
	πMH	5.64±0.04	5.81±0.02	6.34±0.06	5.98±0.06
Co	His	5.93±0.05	5.96±0.05	6.02±0.02	6.90±0.03
	πMH	5.42±0.04	5.57±0.03	5.81±0.03	6.62±0.05

Supplementary Table 17. $k_{cat, ESH+}$ (s^{-1}) values obtained from fitting the k_{cat}-pH profiles to Eq. 2. Mean of three biological replicates. Error values as SEM

M^{2+}	Position 55	Substrate			
		I	II	III	IV
Zn	His	152.5±13.3	67.0±13.6	67.6±9.4	3.3±7.0
	πMH	287.7±18.4	154.3±15.7	90.1±11.0	32.1±10.7
Co	His	543.4±54.9	371.9±37.7	493.5±48.1	79±16
	πMH	1070.2±85.6	746.0±49.0	560.2±63.9	100±14

Supplementary Table 18. $k_{cat, ES}$ (s^{-1}) values obtained from fitting the k_{cat}-pH profiles to Eq. 2. Mean of three biological replicates. Error values as SEM

M^{2+}	Position 55	Substrate			
		I	II	III	IV
Zn	His	2103.0±97.6	1889.4±129.1	2057.9±92.1	741±36
	πMH	2541.1±75.7	2350.7±84.8	2186.7±51.7	455±18
Co	His	5393.0±383.1	5293.3±368.7	6373.5±366.5	2680±95
	πMH	6774.0±343.9	6465.7±253.0	6471.8±309.2	1390±40

Supplementary Table 19. D_2O pK_a^{cat} values for k_{cat} from a fit of the pH-activity data with Eq.2. Mean of three biological replicates. Error values as SEM

M^{2+}	Position 55	Substrate	
		III	IV
Zn	His	6.79±0.09	6.70±0.32
	πMH	6.53±0.09	6.61±0.25
Co	His	7.00±0.05	6.98±0.04
	πMH	6.72±0.06	6.68±0.07

Supplementary Table 20. D_2O pK_a^{cat} values for k_{cat}/K_m from a fit of the pH-activity data with Eq.1. Mean of three biological replicates. Errors values as SEM

M^{2+}	Position 55	Substrate	
		III	IV
Zn	His	6.97±0.06	6.87±0.08
	πMH	6.40±0.06	6.23±0.11
Co	His	6.08±0.03	6.76±0.04
	πMH	5.83±0.03	6.51±0.03

Supplementary Table 21. KSIE values of k_{cat} for substrate III. Mean of three biological replicates. Error values as standard deviation

pH	Variant			
	H55(Zn)	H55πMH(Zn)	H55(Co)	H55πMH(Co)
8.5	2.62±0.24	2.1±0.07	2.19±0.09	1.98±0.26
8.0	2.71±0.30	2.45±0.07	2.42±0.06	2.18±0.32
7.5	2.75±0.32	2.48±0.07	2.43±0.03	2.17±0.32
7.0	2.84±0.25	2.79±0.16	2.64±0.23	2.34±0.28
6.5	2.80±0.27	2.53±0.12	3.21±0.05	2.55±0.35
6.0	2.63±0.30	2.73±0.05	4.07±0.14	2.80±0.30
5.5	3.30±0.18	3.13±0.19	5.39±0.21	3.28±0.33

Supplementary Table 22. KSIE values of k_{cat}/K_m for substrate III. Mean of three biological replicates. Error values as standard deviation

pH	Variant			
	H55(Zn)	H55πMH(Zn)	H55(Co)	H55πMH(Co)
8.5	1.08±0.07	1.17±0.06	1.00±0.02	1.02±0.05
8.0	0.95±0.07	1.05±0.05	0.86±0.03	0.90±0.03
7.5	0.92±0.08	0.94±0.02	0.86±0.02	0.88±0.04
7.0	1.25±0.07	1.17±0.08	1.03±0.02	1.00±0.03
6.5	1.33±0.21	0.98±0.07	0.93±0.01	0.88±0.07
6.0	2.09±0.26	1.54±0.03	0.97±0.03	0.97±0.09
5.5	2.32±0.23	2.13±0.25	0.84±0.01	1.08±0.12

Supplementary Table 23. KSIE values of k_{cat} for substrate IV. Mean of three biological replicates. Error values as standard deviation

pH	Variant			
	H55(Zn)	H55πMH(Zn)	H55(Co)	H55πMH(Co)
8.5	2.47±0.36	1.90±0.15	2.00±0.09	1.76±0.04
8.0	2.31±0.34	1.88±0.14	1.95±0.05	1.70±0.03
7.5	2.19±0.24	1.67±0.10	1.81±0.03	1.46±0.05
7.0	2.08±0.18	1.78±0.10	1.84±0.01	1.52±0.11
6.5	2.45±0.20	1.77±0.32	2.11±0.06	1.79±0.15
6.0	-	1.67±0.19	2.00±0.22	1.72±0.05

Supplementary Table 24. KSIE values of k_{cat}/K_m for substrate IV. Mean of three biological replicates. Error values as standard deviation

pH	Variant			
	H55(Zn)	H55πMH(Zn)	H55(Co)	H55πMH(Co)
8.5	1.20±0.18	1.16±0.06	1.12±0.03	1.21±0.07
8.0	1.27±0.16	1.27±0.04	1.08±0.03	1.15±0.06
7.5	1.07±0.15	1.06±0.01	1.02±0.04	0.99±0.09
7.0	1.23±0.28	1.03±0.10	1.07±0.03	1.07±0.21
6.5	1.37±0.13	1.00±0.13	1.03±0.02	0.97±0.11
6.0	-	1.37±0.13	0.95±0.09	1.00±0.08

Supplementary Table 25. $k_{cat, ESH+}$ (s^{-1}) values obtained from fitting the D_2O k_{cat}-pH profiles to Eq. 2. Mean of three biological replicates. Error values as SEM

M^{2+}	Position 55	Substrate	
		III	IV
Zn	His	32.9±10.7	-
	πMH	43.0±20.9	53.8±27.6
Co	His	148.1±19.8	93.9±13.9
	πMH	395.0±44.3	69.2±23.5

Supplementary Table 26. $k_{cat, ES}$ (s^{-1}) values obtained from fitting the D_2O k_{cat}-pH profiles to Eq. 2. Mean of three biological replicates. Error values as SEM

M^{2+}	Position 55	Substrate	
		III	IV
Zn	His	781.3±51.2	326.6±25.5
	πMH	943.5±51.6	255.5±17.8
Co	His	3161.4±140.0	1453.9±33.5
	πMH	3417.6±121.5	865.5±22.8

Supplementary Table 27. πMH/His KSIE ratios for k_{cat} and k_{cat}/K_m values for substrate III. Mean of three biological replicates. Error values as standard deviation

pH	Zn		Co	
	k_{cat}	k_{cat}/K_m	k_{cat}	k_{cat}/K_m
8.5	0.80±0.03	1.08±0.05	0.90±0.12	1.02±0.05
8.0	0.90±0.03	1.11±0.05	0.90±0.13	1.05±0.03
7.5	0.90±0.02	1.02±0.02	0.89±0.13	1.02±0.05
7.0	0.98±0.06	0.94±0.06	0.89±0.11	0.96±0.03
6.5	0.90±0.04	0.74±0.05	0.79±0.11	0.94±0.07
6.0	1.04±0.02	0.74±0.01	0.69±0.07	0.99±0.09
5.5	0.95±0.06	0.92±0.11	0.61±0.06	1.28±0.14

Supplementary Table 28. πMH/His KSIE ratios for k_{cat} and k_{cat}/K_m values for substrate IV. Mean of three biological replicates. Error values as standard deviation

pH	Zn		Co	
	k_{cat}	k_{cat}/K_m	k_{cat}	k_{cat}/K_m
8.5	0.77±0.06	0.97±0.05	0.88±0.02	1.07±0.06
8.0	0.82±0.06	1.00±0.03	0.87±0.02	1.06±0.06
7.5	0.76±0.05	0.99±0.01	0.80±0.03	0.98±0.08
7.0	0.86±0.05	0.84±0.09	0.82±0.06	1.00±0.20
6.5	0.72±0.13	0.73±0.09	0.85±0.07	0.94±0.10
6.0	-	-	0.86±0.02	1.05±0.09

Supplementary Table 29. KSIE values for $k_{cat, ESH+}$ (s^{-1}) obtained from fitting the k_{cat}-pH profiles to Eq. 2. Mean of three biological replicates. Error values as SEM

M^{2+}	Position 55	Substrate	
		III	IV
Zn	His	2.1±0.4	-
	πMH	2.1±0.5	0.6±0.6
Co	His	3.3±0.2	0.4±0.3
	πMH	1.4±0.2	0.3±0.6

Supplementary Table 30. ΔpK_a^{cat} (D_2O - H_2O) values for the k_{cat} pK_a^{cat} values obtained in H_2O and D_2O. Mean of three biological replicates. Error values as SEM

**Substrate**		**Substrate**			
		H55(Zn)	H55πMH(Zn)	H55(Co)	H55πMH(Co)
III	k_{cat}	0.103±0.078	0.175±0.086	0.487±0.087	0.535±0.087
	k_{cat}/K_m	0.214±0.096	0.053±0.087	0.059±0.037	0.022±0.060
IV	k_{cat}	0.04±0.11	0.20±0.12	0.143±0.068	0.056±0.079
	k_{cat}/K_m	0.152±0.092	0.255±0.081	0.143±0.045	0.109±0.063

Supplementary Table 31. KSIE values for $k_{cat, ES}$ (s^{-1}) obtained from fitting the k_{cat}-pH profiles to Eq. 2. Mean of three biological replicates. Error values as SEM

**M^{2+}**	**Position 55**	**Substrate**	
		III	IV
Zn	His	2.6±0.1	2.3±0.1
	πMH	2.3±0.1	1.8±0.1
Co	His	2.0±0.1	1.8±0.1
	πMH	1.9±0.1	1.5±0.1

Supplementary Table 32. Values obtained from the Brønsted analysis at pH 8.5 from a fit to Eq. 6-8. Mean of three biological replicates. Error values as SEM

**M^{2+}**	**Position**	**Parameter**				
	**55**	k_1 ($s^{-1}\mu M^{-1}$)	k_2 (s^{-1})	k_5 (s^{-1})	β	C
Zn	His	18.8±1.8	3539.3±634.1	2316.8±115.4	-1.2±0.1	12.9±1.1
	πMH	17.0±1.6	3375.9±556.5	2767.1±113.7	-1.3±0.1	13.4±0.7
Co	His	16.6±1	1118.5±249.6	6858.1±235.5	-1.9±0.1	18.8±1.0
	πMH	16.0±1.1	1405.2±233.8	7615.3±226.7	-1.7±0.1	17.0±0.6

Supplementary Table 33. Values obtained from the Brønsted analysis at pH 8.0 from a fit to Eq. 6-8. Mean of three biological replicates. Error values as SEM

**M^{2+}**	**Position**	**Parameter**				
	**55**	k_1 ($s^{-1}\mu M^{-1}$)	k_2 (s^{-1})	k_5 (s^{-1})	β	C
**Zn**	**His**	17.5±1.9	3657.2±741.6	1988.5±104.0	-1.2±0.2	13.0±1.3
	**πMH**	16.8±2.5	4725.2±1197.9	2480.8±159.6	-1.1±0.1	11.8±1.0
**Co**	**His**	16.5±1.0	1455.0±288.4	5906.3±235.9	-2.0±0.2	20.2±1.3
	**πMH**	15.6±1.0	1449.5±228.8	6867.2±244.5	-1.8±0.1	17.9±0.8

Supplementary Table 34. Values obtained from the Brønsted analysis at pH 7.5 from a fit to Eq. 6-8. Mean of three biological replicates. Error values as SEM.

**M^{2+}**	**Position**	**Parameter**				
	**55**	k_1 ($s^{-1}\mu M^{-1}$)	k_2 (s^{-1})	k_5 (s^{-1})	β	C
Zn	His	18.0±3.0	4310.3±1287.3	1754.7±134.6	-0.9±0.2	10.2±1.4
	πMH	17.7±3.1	4744.2±1358.2	2350.1±231.0	-0.9±0.1	10.4±1.2
Co	His	15.6±1.2	1286.7±309.7	4612.1±216.9	-1.7±0.2	17.7±1.5
	πMH	15.2±1.0	1405.1±225.4	6435.5±246.0	-1.5±0.1	15.5±0.7

Supplementary Table 35. Individual distances measured in the crystal structures in Å.

Variant	H55		H55πMH					
Chain ID	A	B	A	B	C	D	E	F
Zn-Zn	3.6	3.7	3.6	3.6	3.4	3.3	3.4	3.3
Zn(α)-μH_2O	2.1	2.2	2.1	2.1	2.2	2.1	2.2	2.3
Zn(β)-μH_2O	2.3	2.5	2.2	2.2	2.2	2.2	2.1	2.7
Asp301-μH_2O	2.8	2.5	2.6	2.5	2.7	2.5	2.3	2.2
His/πMH55-Zn(α)	2.2	2.1	2.3	2.2	1.9	2.1	2.1	2.0
His57-Zn(α)	2.1	2.2	2.3	2.1	2.2	2.2	2.1	2.2
Lys169-Zn(α)	2.4	2.4	2.4	2.3	2.5	2.5	2.5	2.5
Lys169-Zn(β)	2.2	2.2	1.9	2.3	2.3	2.3	2.4	2.4
His201-Zn(β)	2.1	2.1	2.1	2.1	2.2	2.2	2.1	2.3
His230-Zn(β)	2.1	2.1	2.1	2.3	2.2	2.2	2.2	2.2
Asp301-Zn(α)	2.2	2.2	2.3	2.4	2.4	2.4	2.4	2.5

Supplementary Table 36. Primers used for dPTE2 variant cloning

Name	Sequence 5'→3'
dPTE2-H55tag-For	CACTCTGATGTAGGAGCATATCTG
dPTE2-H55tag-Rev	AAACCGGCCTCAGAG

Supplementary Table 37. Crystallographic data.

	dPTE2-H55	dPTE2-H55πMH
Data collection:		
Wavelength (Å)	0.9677	0.9677
Space group	P $2_1 2_1 2_1$	C 1 2 1
a, b, c (Å)	63.49, 69.98, 154.16	442.55, 57.93, 84.46
α, β, γ (°)	90, 90, 90	90, 100.88, 90
Resolution (Å) *	19.69 – 1.55 (1.72 – 1.55)	19.90 – 1.69 (1.87 – 1.69)
R_{meas}*	0.289 (2.048)	0.164 (1.330)
R_{merge}*	0.278 (1.976)	0.152 (1.235)
Mean I/σI*	6.9 (1.4)	8.2 (1.5)
Completeness ellipsoidal (%)*	93.8 (44.5)	90.5 (61.5)
Multiplicity*	13.8 (14.3)	7.1 (7.3)
CC1/2*	0.994 (0.599)	0.996 (0.658)
Refinement:		
Resolution (Å)	19.69 – 1.55	19.90 – 1.69
Total reflections*	1061968 (55076)	998498 (50845)
Total unique*	76985 (3849)	139776 (6989)
R_{work}#	0.1775 (0.2714)	0.2039 (0.2244)
R_{free}#	0.1872 (0.2741)	0.2164 (0.3512)
Number of non-hydrogen atoms	5466	15830
macromolecules	5051	15069
ligands	8	60
solvent	407	701
Protein residues	655	1956
RMS deviations (bonds)#	0.008	0.008
RMS deviations (angles)#	0.89	0.92
Ramachandran favored (%)#	97.67	96.91
Ramachandran allowed (%)#	2.33	3.09
Ramachandran outliers (%)#	0.00	0.00
Rotamer outliers (%)#	0.76	0.51
Clashscore#	1.49	2.20
Average B-factor#	14.75	26.14
macromolecules	14.33	26.29
ligands	18.37	26.21
solvent	19.91	23.05
Molprobity Score	0.96	1.18
PDB	9TI1	9TI2

#as reported by phenix.table_one
*as reported by autoPROC and phenix.table_one

III. Chemical synthesis

General procedure for the synthesis of paraoxon analogues I, II, IV, V.

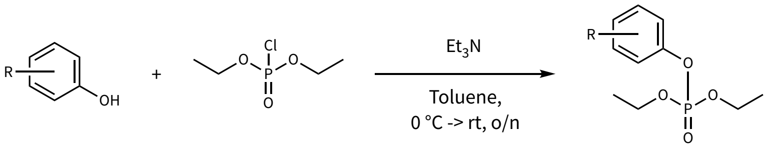

Procedure was adapted from literature.[1]

The phenol (1.0 eq.) was dissolved in toluene under an N_2 atmosphere and cooled to 4 °C using an ice-water bath. Diethyl chlorophosphate (1.5 eq.) was added dropwise, followed by the dropwise addition of Et_3N (1.5 eq.). The reaction was allowed to warm to room temperature. and stirred overnight. The reaction was transferred to a separatory funnel and washed with water (4x). The organic layer was dried over $MgSO_4$ and concentrated *in vacuo*. The crude was purified by automated flash chromatography (SiO_2, EtOAc in cyclohexane).

2,6-Difluoro-4-nitrophenyl diethyl phosphate (I)

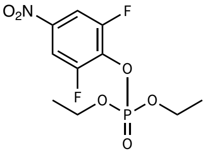

Purification using 30% EtOAc in cyclohexane to yield 2,6-difluoro-4-nitrophenyl diethyl phosphate (941 mg, 3.0 mmol, 53%) as a yellow oil.

^{1}H-NMR (400 MHz, $CDCl_3$) δ 7.94-7.89 (m, 2H), 4.37-4.28 (m, 4H), 1.43-1.39 (m, 6H).

Lit:[2] ^{1}H NMR (400 MHz, $CDCl_3$) δ 7.94 (d, 2H, J = 7.16 Hz), 4.40-4.30 (m, 4H), 1.44 (td, 6H, J_1 = 7.16 Hz, J_2 = 1.10 Hz).

^{13}C-NMR (101 MHz, $CDCl_3$) δ 155.90 (t, J = 4.1 Hz), 153.36 (t, J = 4.0 Hz), 144.27-143.72 (m), 109.14-108.61 (m), 65.77 (d, J = 6.3 Hz), 16.02 (d, J = 7.1 Hz).

3-Fluoro-4-nitrophenyl diethyl phosphate (II)

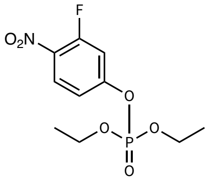

Purification using 50% EtOAc in cyclohexane to yield 3-fluoro-4-nitrophenyl diethyl phosphate (1.0 g, 3.4 mmol, 54%) as a dark-yellow oil.

^{1}H-NMR (400 MHz, $CDCl_3$) δ 8.10 (t, J = 8.7 Hz, 1H), 7.23-7.15 (m, 2H), 4.29-4.21 (m, 4H), 1.40-1.36 (m, 6H).

Lit:[2] ^{1}H NMR (400 MHz, $CDCl_3$) δ 8.13 (t, 1H, J = 8.84 Hz), 7.28-7.18 (m, 2H), 4.32-4.24 (m, 4H), 1.41 (td, 6H, J_1 = 7.10 Hz, J_2 = 1.04 Hz).

^{13}C-NMR (101 MHz, $CDCl_3$) δ 157.85, 155.19, 134.20 (d, J = 6.8 Hz), 127.77 (d, J = 1.8 Hz), 116.24-116.14 (m), 110.57-110.17 (m), 65.52 (d, J = 6.2 Hz), 16.14 (d, J = 6.5 Hz).

4-Acetylphenyl diethyl phosphate (IV)

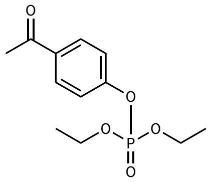

Purification using 50% EtOAc in cyclohexane to yield 4-acetylphenyl diethyl phosphate as a pale-yellow oil. Yield was not determined, due to only a fraction of the crude being purified.

^{1}H-NMR (400 MHz, $CDCl_3$) δ 7.93 (d, J = 8.3 Hz, 2H), 7.27 (d, J = 8.3 Hz, 2H), 4.24-4.16 (m, 4H), 2.55 (s, 3H), 1.33 (t, J = 7.2 Hz, 6H).

Lit:[2] ^{1}H NMR (400 MHz, $CDCl_3$) δ 7.98 (d, 2H, J = 8.75 Hz), 7.32 (dd, 2H, J_1 = 8.75 Hz, J_2 = 0.90 Hz) 4.30-4.21 (m, 4H), 2.60 (s, 3H), 1.38 (td, 6H, J_1 = 7.10 Hz, J_2 = 0.98 Hz).

^{13}C-NMR (101 MHz, $CDCl_3$) δ 196.75, 154.50 (d, J = 6.7 Hz), 133.99, 130.41, 119.98 (d, J = 5.4 Hz), 64.94 (d, J = 6.2 Hz), 26.58, 16.11 (d, J = 6.6 Hz).

Methyl 4-((diethoxyphosphoryl)oxy)benzoate (V)

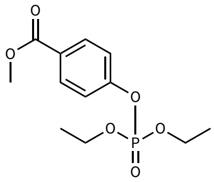

Purification using 30% EtOAc in cyclohexane to yield methyl 4-((diethoxyphosphoryl) oxy)benzoate (1.1 g, 3.8 mmol, 58%) as a pale-yellow oil.

^{1}H-NMR (400 MHz, $CDCl_3$) δ 8.04-8.01 (m, 2H), 7.28-7.26 (m, 2H), 4.24-4.20 (m, 4H), 3.90 (s, 3H), 1.37-1.33 (m, 6H).

Lit:[2] ^{1}H NMR (400 MHz, $CDCl_3$) δ 8.05 (d, 2H, J = 8.75 Hz), 7.29 (d, 2H, J = 8.75 Hz) 4.30-4.15 (m, 4H), 3.93 (s, 3H), 1.34 (td, 6H, J_1 = 7.20 Hz, J_2 = 0.98 Hz).

^{13}C-NMR (101 MHz, $CDCl_3$) δ 166.38, 154.50 (d, J = 6.5 Hz), 131.68, 127.00, 119.93 (d, J = 5.3 Hz), 65.00 (d, J = 6.1 Hz), 52.29, 16.19 (d, J = 6.7 Hz)..

IV. NMR spectra

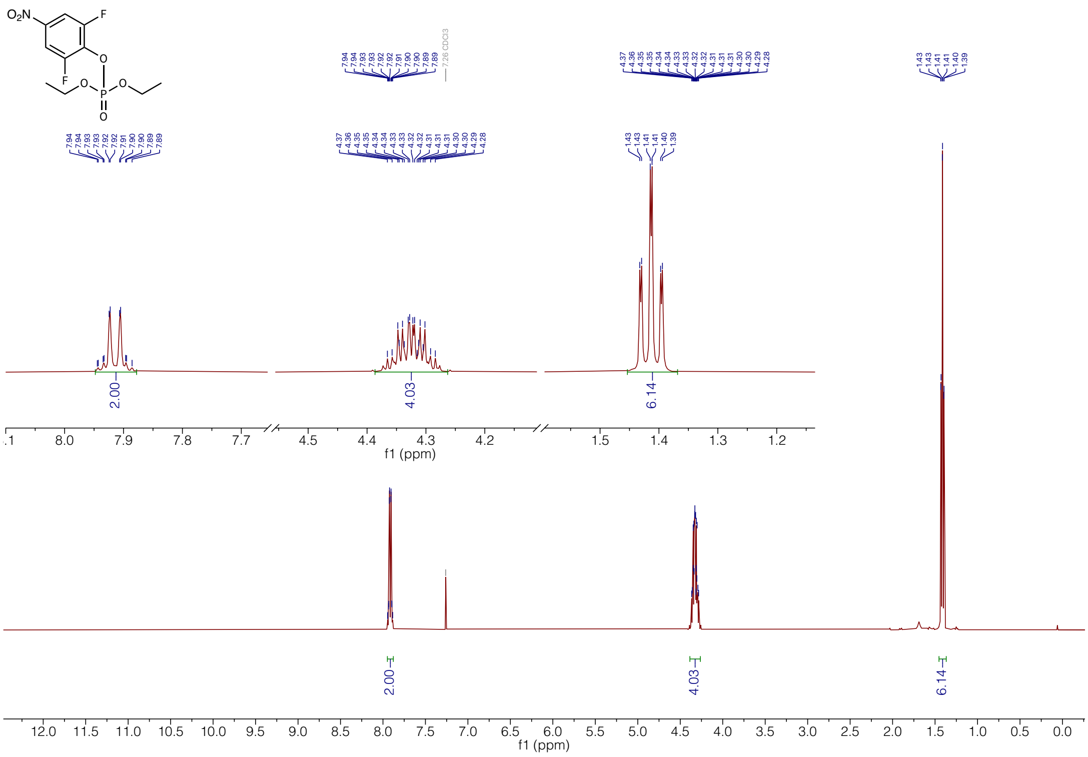

Supplementary Figure 17. ^{1}H-NMR spectrum of 2,6-Difluoro-4-nitrophenyl diethyl phosphate (I)

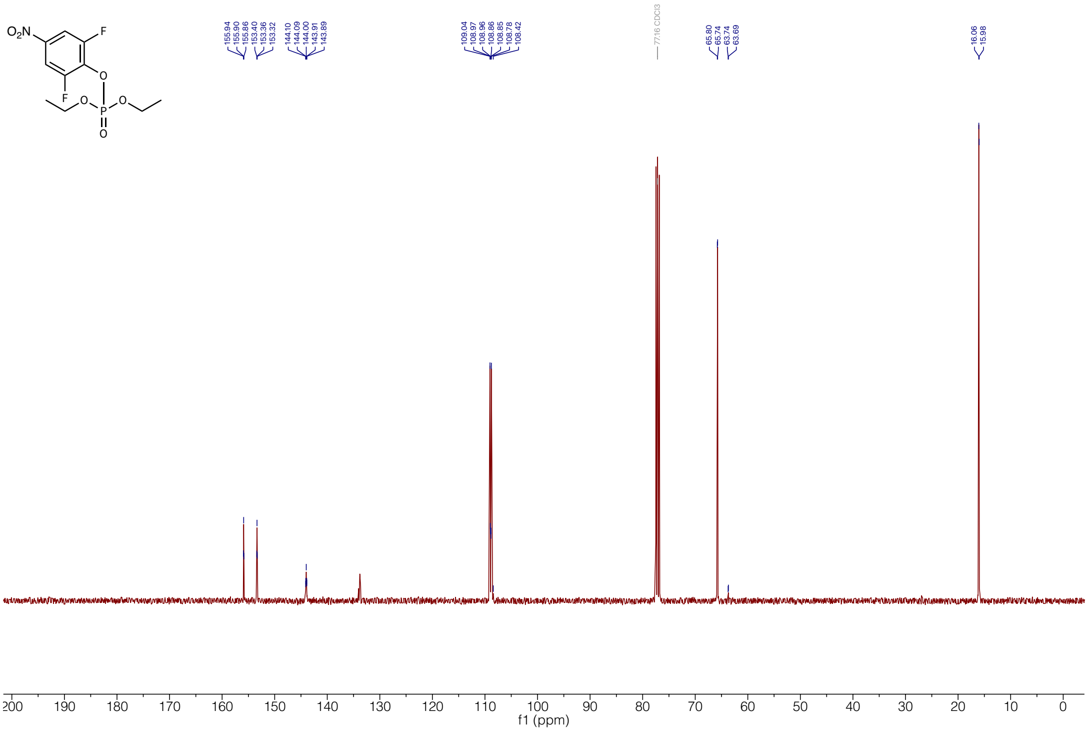

Supplementary Figure 18. ^{13}C-NMR spectrum of 2,6-Difluoro-4-nitrophenyl diethyl phosphate (I)

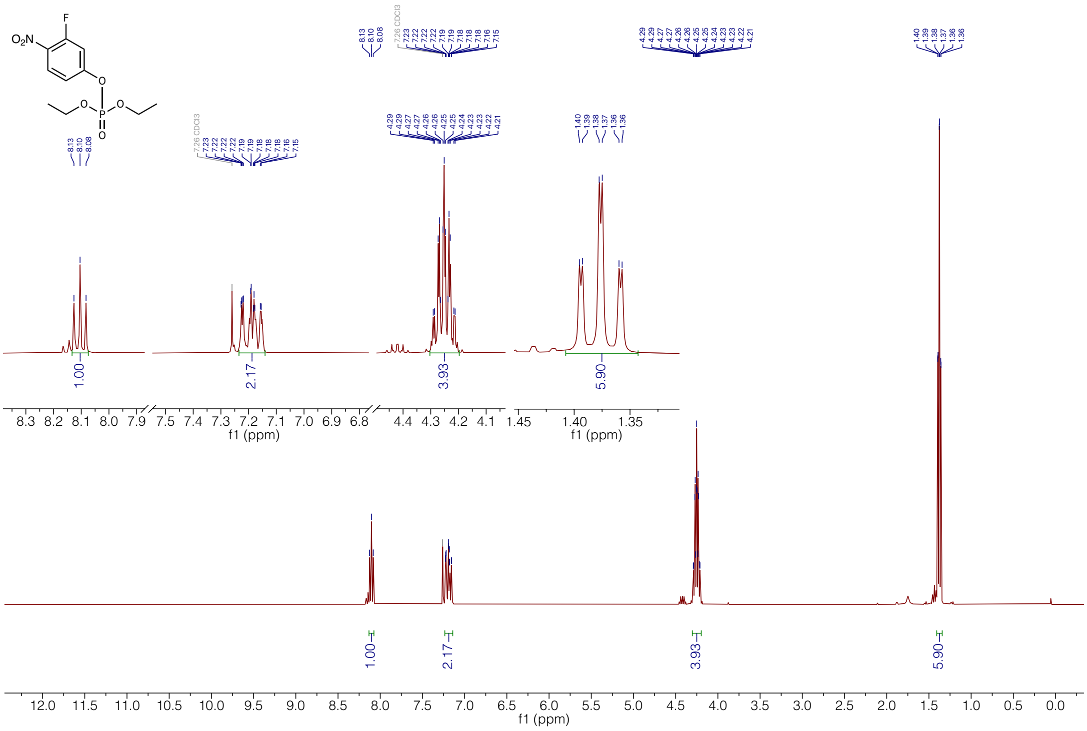

Supplementary Figure 19. ^{1}H-NMR spectrum of 3-Fluoro-4-nitrophenyl diethyl phosphate (II)

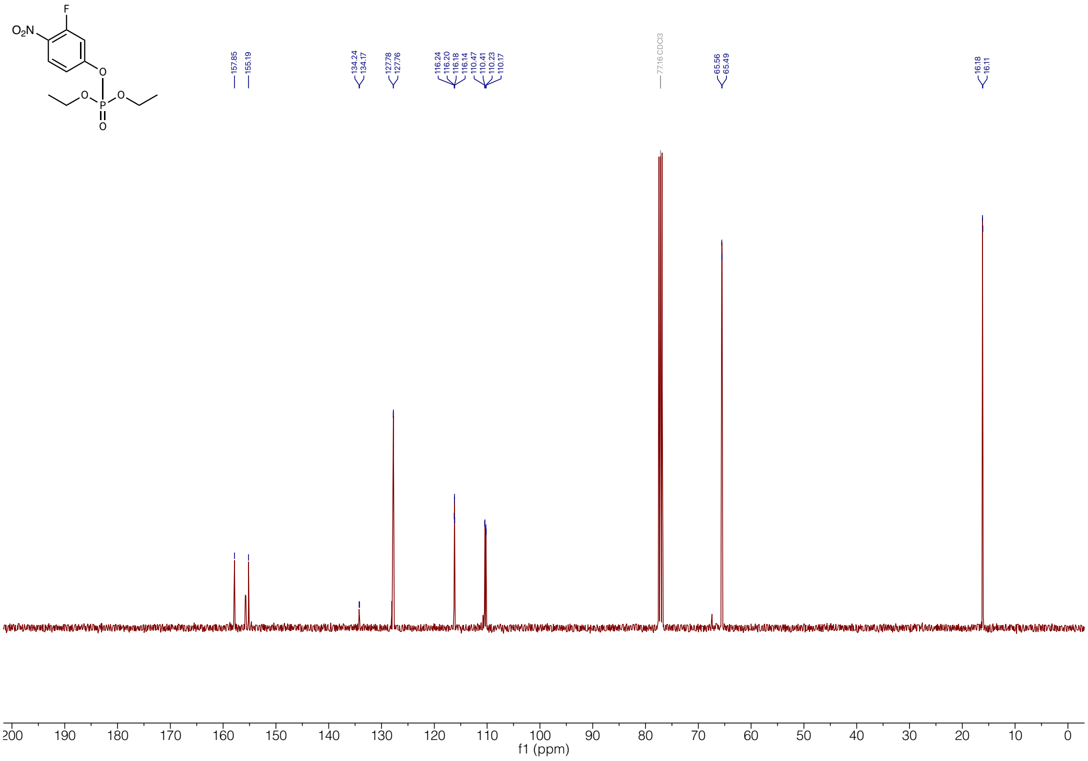

Supplementary Figure 20.^{13}C-NMR spectrum of 3-Fluoro-4-nitrophenyl diethyl phosphate (II)

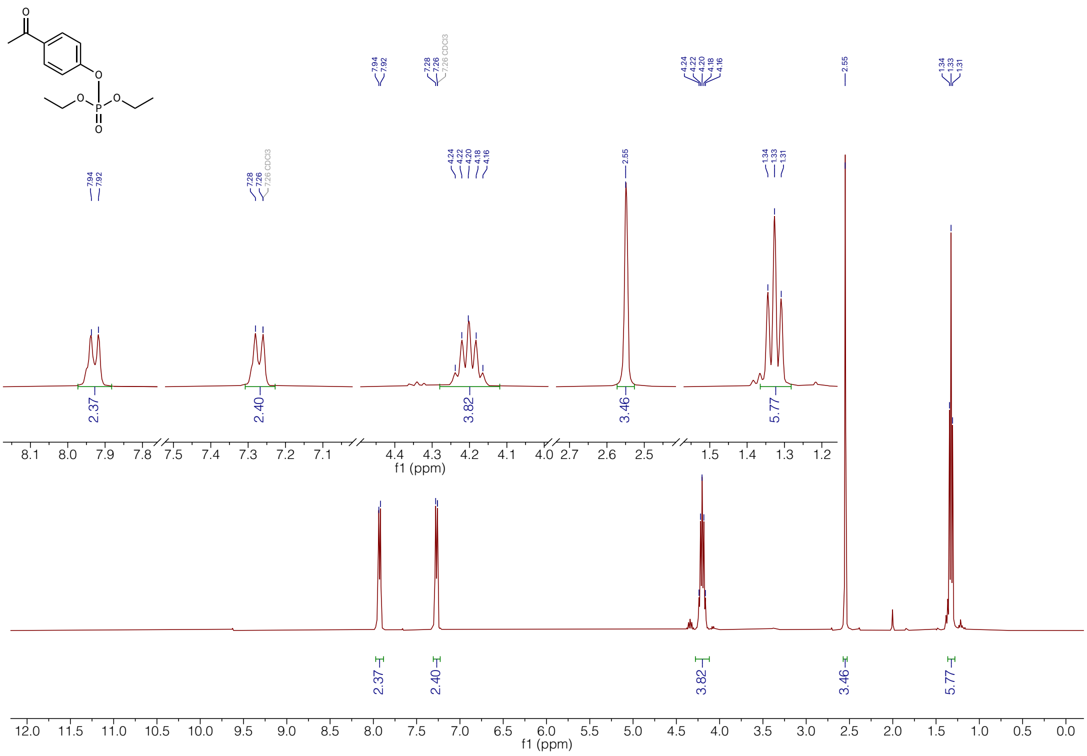

Supplementary Figure 21.^{1}H-NMR spectrum of 4-Acetylphenyl diethyl phosphate (IV)

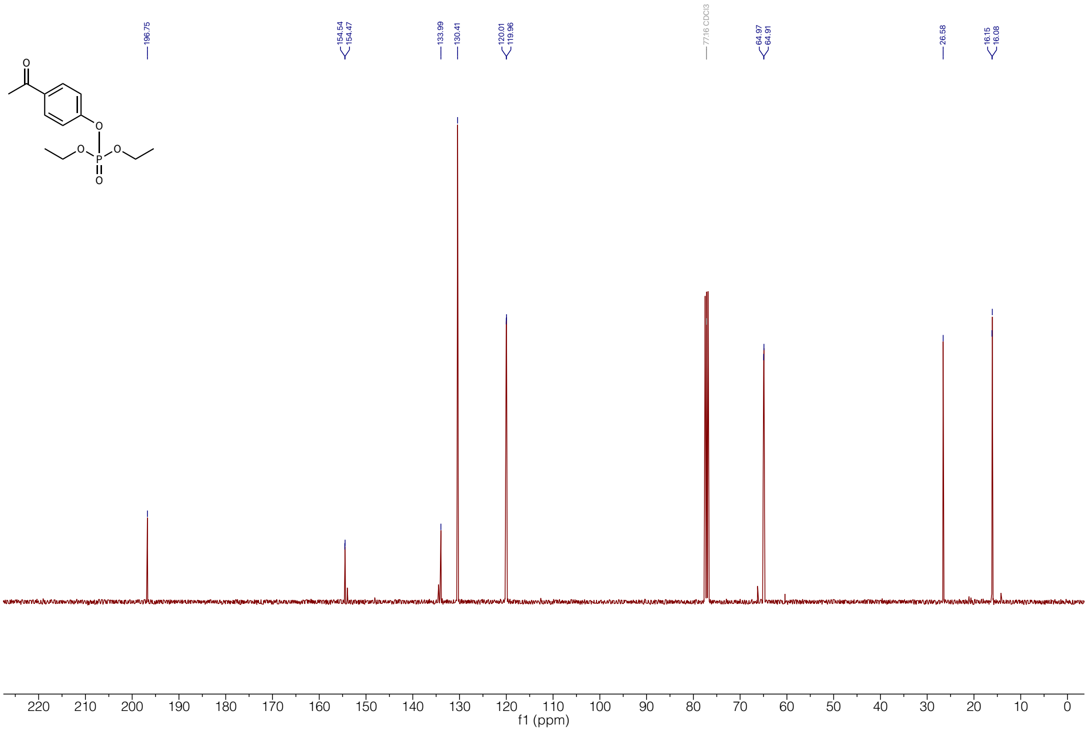

Supplementary Figure 22.^{13}C-NMR spectrum of 4-Acetylphenyl diethyl phosphate (IV)

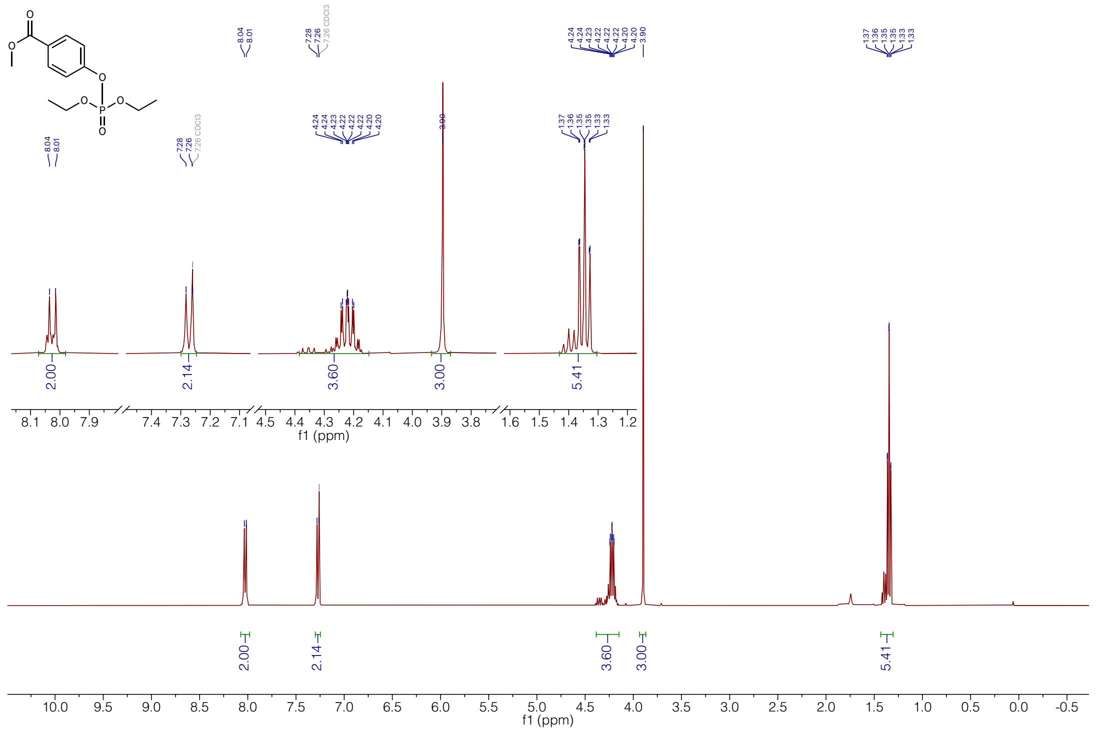

Supplementary Figure 23.^{1}H-NMR spectrum of Methyl 4-((diethoxyphosphoryl)oxy)benzoate (V)

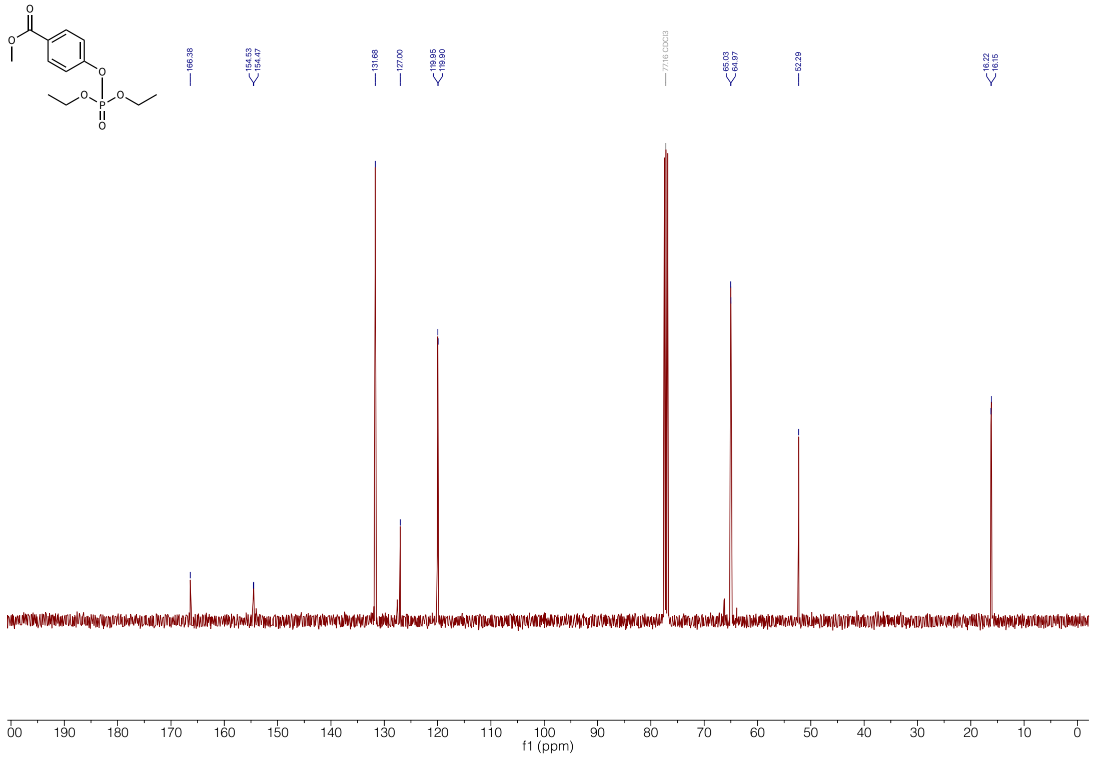

Supplementary Figure 24. ^{13}C-NMR spectrum of Methyl 4-((diethoxyphosphoryl)oxy)benzoate (V)

V. DNA and Protein sequences

DNA sequence 1. strepTagII-SUMO-(tev)-dPTE2-H55

ATGGGTTCTTCTTGGAGCCACCCGCAGTTCGAAAAAGGTTCTTCTATGGCTAGCATGTCCGACTCTGA
AGTCAATCAAGAAGCTAAGCCAGAGGTCAAGCCAGAAGTCAAGCCTGAGACTCACATCAATTTAAAGG
TGTCCGATGGATCTTCTGAGATCTTCTTCAAGATCAAAAAGACCACTCCTTTAAGAAGGCTGATGGAA
GCGTTCGCTAAAAGACAGGGTAAGGAAATGGACTCCTTAAGATTCTTGTACGACGGTATTAGAATTCA
AGCTGATCAGACCCCTGAAGATTTGGACATGGAGGATAACGATATTATTGAGGCTCACAGAGAACAGA
TTGGTGGGATCGAGGAAAACCTGTACTTCCAATCCAATATTGGAAGTGGAATCACCAACAGCGGCGAC
CGTATCAACACCGTTCGTGGTCCGATCACCATCTCTGAGGCCGGTTTCACTCTGATGCATGAGCATAT
CTGCGGTTCTTCTGCGGGTTTTCTCCGCGCTTGGCCGGAGTTCTTCGGTTCCCGTGATGCACTGGCGG
AAAAAGCGGTTCGCGGCCTCCGTCGCGCTCGTGCTGCCGGCGTTCGTACCATCGTTGATGTTTCTACC
TTTGATATCGGCCGTGACGTTGAGCTGCTCGCCGAAGTTTCTGAAGCGGCGGACGTTCACATCGTCGC
GGCGACGGGTCTGTGGTTCGACCCGCCTCTGTCTATGCGCCTGCGTTCTGTTGAGGAACTCACCCAGT
TCTTTCTGCGCGAAATCCAGTACGGTATCGAGGATACCGGCATCCGTGCAGGTATCATCAAAGTTGCG
ACCACCGGTAAAGCCACGCCGTTCCAGGAGCGTGTACTCCGTGCGGCTGCACGCGCCTCTCTCGCGAC
CGGTGTGCCTGTTACCACCCATACCGATGCTTCTCAGCGTGACGGTGAGCAGCAGGCGGACATCTTCG
AATCTGAAGGCCTCGATCCGAGCCGTGTTTGCATTGGTCATTCTGACGACACCGACGACCTGGACTAC
CTGACCGCTCTCGCAGCTCGCGGTTATCTGATTGGTCTGGATCACATTCCGCACAGCGCGATCGGCCT
GGAAGACAACGCGTCCGCTGCCGCGCTGCTGGGCCTGCGCTCTTGGCAAACCCGTGCCCTCCTGATCA
AAGCGCTGATCGACCAGGGCTACGCCGACCAGATCCTGGTTTCTAACGACTGGCTGTTCGGTTTCTCT
TCTTACGTTACCAACATCATGGATGTCATGGACCGTGTTAACCCGGATGGTATGGCGTTCATCCCTCT
GCGTGTTATTCCATTCCTCCGTGAAAAAGGTGTTCCGGACGAGACTCTCGAAACGATCATGGTTGACA
ACCCAGCTCGTTTCCTGTCTCCGACCCTGCGTGCGAGCTAA

DNA sequence 2. strepTagII-SUMO-(tev)-dPTE2-H55TAG

ATGGGTTCTTCTTGGAGCCACCCGCAGTTCGAAAAAGGTTCTTCTATGGCTAGCATGTCCGACTCTGA
AGTCAATCAAGAAGCTAAGCCAGAGGTCAAGCCAGAAGTCAAGCCTGAGACTCACATCAATTTAAAGG
TGTCCGATGGATCTTCTGAGATCTTCTTCAAGATCAAAAAGACCACTCCTTTAAGAAGGCTGATGGAA
GCGTTCGCTAAAAGACAGGGTAAGGAAATGGACTCCTTAAGATTCTTGTACGACGGTATTAGAATTCA
AGCTGATCAGACCCCTGAAGATTTGGACATGGAGGATAACGATATTATTGAGGCTCACAGAGAACAGA
TTGGTGGGATCGAGGAAAACCTGTACTTCCAATCCAATATTGGAAGTGGAATCACCAACAGCGGCGAC
CGTATCAACACCGTTCGTGGTCCGATCACCATCTCTGAGGCCGGTTTCACTCTGATGTAGGAGCATAT
CTGCGGTTCTTCTGCGGGTTTTCTCCGCGCTTGGCCGGAGTTCTTCGGTTCCCGTGATGCACTGGCGG
AAAAAGCGGTTCGCGGCCTCCGTCGCGCTCGTGCTGCCGGCGTTCGTACCATCGTTGATGTTTCTACC
TTTGATATCGGCCGTGACGTTGAGCTGCTCGCCGAAGTTTCTGAAGCGGCGGACGTTCACATCGTCGC
GGCGACGGGTCTGTGGTTCGACCCGCCTCTGTCTATGCGCCTGCGTTCTGTTGAGGAACTCACCCAGT
TCTTTCTGCGCGAAATCCAGTACGGTATCGAGGATACCGGCATCCGTGCAGGTATCATCAAAGTTGCG
ACCACCGGTAAAGCCACGCCGTTCCAGGAGCGTGTACTCCGTGCGGCTGCACGCGCCTCTCTCGCGAC
CGGTGTGCCTGTTACCACCCATACCGATGCTTCTCAGCGTGACGGTGAGCAGCAGGCGGACATCTTCG
AATCTGAAGGCCTCGATCCGAGCCGTGTTTGCATTGGTCATTCTGACGACACCGACGACCTGGACTAC
CTGACCGCTCTCGCAGCTCGCGGTTATCTGATTGGTCTGGATCACATTCCGCACAGCGCGATCGGCCT
GGAAGACAACGCGTCCGCTGCCGCGCTGCTGGGCCTGCGCTCTTGGCAAACCCGTGCCCTCCTGATCA
AAGCGCTGATCGACCAGGGCTACGCCGACCAGATCCTGGTTTCTAACGACTGGCTGTTCGGTTTCTCT
TCTTACGTTACCAACATCATGGATGTCATGGACCGTGTTAACCCGGATGGTATGGCGTTCATCCCTCT
GCGTGTTATTCCATTCCTCCGTGAAAAAGGTGTTCCGGACGAGACTCTCGAAACGATCATGGTTGACA
ACCCAGCTCGTTTCCTGTCTCCGACCCTGCGTGCGAGCTAA

DNA sequence 3. pOS1T-G1PylRS^{MIFAF}-PylT$^{G1}_{CUA}$

The pOS1T-G1PylRS^{MIFAF}-PylT$^{G1}_{CUA}$ plasmid contains a p15A type origin of replication, a tetracycline resistance gene (Tet^{R}), the G1PylRS$^{MIFAF.}$ under control of an OXB20 promoter, and a PylT$^{G1}_{CUA}$ under control of a proK promoter.

GCACCATTCCTTGCGGCGGCGGTGCTCAACGGCCTCAACCTACTACTGGGCTGCTTCCTAATGCAGGA
GTCGCATAAGGGAGAGCGTCTGGCGAAAAAAAAGCCTGCTCGTTGAGCAGGCTTTTCGAATTTGGCGA
AAGGCCGGGGGGTCGAACCCCGCTTACAGGTTTTAGAGACCCGTTTGCTCGCCGGAGCGCCCTCCAAT
GCGGGGCGCATCTTACTGCGCAGATACGCCCTCGTCAATCCCTTAATAGCAAAATGCCTCCTGCACCA
TTATGTTCCGGATCTGCAAAGCTGTTGTGACCGCTTGCTCTAGCCAGCTATCGAGTTGTGAACCGATC
CATCTAGCAATTGGTCTCGATCTAGCGATAGGCTTCGATCTAGCTATGTAGAAACGCCGTGTGCTCGA
TCGCTTGATAAGGTCCACGTAGCTGCTATAATTGCTTCAACAGAACATATTGACTATCCGGTATTACC
CGGCCGCCGTTATACGTTGTTTACGCTTTGAGGAATCCCATATGGTGGTCAAGTTTACTGATTCCCAA
ATTCAACACCTTATGGAGTATGGCGATAATGATTGGTCAGAGGCCGAATTCGAAGACGCTGCTGCACG
CGACAAAGAGTTCTCCAGTCAATTTTCCAAACTGAAGTCTGCAAATGATAAAGGGCTGAAAGACGTTA
TTGCCAACCCTCGCAACGATTTAACCGACCTTGAAAACAAGATCCGTGAAAAACTTGCAGCGCGCGGT
TTTATTGAGGTGCACACTCCCATCTTTGTTAGTAAGTCAGCCTTAGCGAAAATGACTATTACCGAGGA
CCACCCGCTTTTTAAGCAAGTTTTTTGGATCGATGATAAACGTGCGTTGCGTCCGATGATGGCTATGA
ATATCTTTAAAGTTGCTCGCGAGTTACGCGATCACACCAAAGGCCCGGTTAAGATTTTTGAGATCGGT
TCGTGCTTTCGCAAAGAGTCCAAGTCATCTACCCATTTGGAGGAGTTTACAATGTTAAATCTGTTTGA
AATGGGGCCAGACGGGGATCCTATGGAGCATCTTAAAATGTACATTGGGGACATCATGGACGCGGTAG
GCGTTGAATACACGACCTCCCGCGAAGAAAGTGATGTATATGTTGAAACCTTGGATGTGGAAATCAAT
GGTACAGAGGTAGCCAGTGGCGCTGTCGGCCCCCACAAATTAGACCCCGCGCACGACGTGCATGAGCC
TTGGGCAGGTATCGGTTTCGGGCTTGAGCGCTTACTTATGCTTAAAAATGGAAAATCAAATGCGCGTA
AGACCGGGAAATCTATCACTTATCTTAACGGATACAAGTTAGACTAACTGCAGTTTCAAACGGGTACC
ATATGGGAATTCGAAGCTTGGGCCCGAACAAAAACTCATCTCAGAAGAGGATCTGAATAGCGCCGTCG
ACCATCATCATCATCATCATTGAGTTTAAACGGTCTCCAGCTTGGCTGTTTTGGCGGATGAGAGAAGA
TTTTCAGCCTGATACAGATTAAATCAGAACGCAGAAGCGGTCTGATAAAACAGAATTTGCCTGGCGGC
AGTAGCGCGGTGGTCCCACCTGACCCCATGCCGAACTCAGAAGTGAAACGCCGTAGCGCCGATGGTAG
TGTGGGGTCTCCCCATGCGAGAGTAGGGAACTGCCAGGCATCAAATAAAACGAAAGGCTCAGTCGAAA
GACTGGGCCTTTCGTTTTATCTGTTGTTTGTCGGTGAACGCTCTCCTGAGTAGGACAAATCCGCCGGG
AGCTGTCCCTCCTGTTCAGCTACTGACGGGGTGGTGCGTAACGGCAAAAGCACCGCCGGACATCAGCG
CTAGCGGAGTGTATACTGGCTTACTATGTTGGCACTGATGAGGGTGTCAGTGAAGTGCTTCATGTGGC
AGGAGAAAAAAGGCTGCACCGGTGCGTCAGCAGAATATGTGATACAGGATATATTCCGCTTCCTCGCT
CACTGACTCGCTACGCTCGGTCGTTCGACTGCGGCGAGCGGAAATGGCTTACGAACGGGGCGGAGATT
TCCTGGAAGATGCCAGGAAGATACTTAACAGGGAAGTGAGAGGGCCGCGGCAAAGCCGTTTTTCCATA
GGCTCCGCCCCCCTGACAAGCATCACGAAATCTGACGCTCAAATCAGTGGTGGCGAAACCCGACAGGA
CTATAAAGATACCAGGCGTTTCCCCCTGGCGGCTCCCTCGTGCGCTCTCCTGTTCCTGCCTTTCGGTT
TACCGGTGTCATTCCGCTGTTATGGCCGCGTTTGTCTCATTCCACGCCTGACACTCAGTTCCGGGTAG
GCAGTTCGCTCCAAGCTGGACTGTATGCACGAACCCCCCGTTCAGTCCGACCGCTGCGCCTTATCCGG
TAACTATCGTCTTGAGTCCAACCCGGAAAGACATGCAAAAGCACCACTGGCAGCAGCCACTGGTAATT
GATTTAGAGGAGTTAGTCTTGAAGTCATGCGCCGGTTAAGGCTAAACTGAAAGGACAAGTTTTGGTGA
CTGCGCTCCTCCAAGCCAGTTACCTCGGTTCAAAGAGTTGGTAGCTCAGAGAACCTTCGAAAAACCGC
CCTGCAAGGCGGTTTTTTCGTTTTCAGAGCAAGAGATTACGCGCAGACCAAAACGATCTCAAGAAGAT
CATCTTATTAATCAGATAAAATATTTCTAGATTTCAGTGCAATTTATCTCTTCAAATGTAGCACCTGA
AGTCAGCCCCATACGATATAAGTTGTAATTCTCATGTTGATCGGCACGTAATAGCGGGGCGTAAATGG
AAGCCGGCGGCGCTGGTAGCAAAATTCCTCGACGAACGTGGGATCGTAGTAGAGAAAACCGGCCCTTA
TAACCTGCTGTTTCTCTTTAGTATTGGCATCGATAAAACCAAAGCAATGGGATTATTGCGTGGGTTGA
CGGAATTCAAACGCTCTTACGATCTCAACCTGCGGATCAAAAATATGCTACCCGATCTCTATGCAGAA
GATCCCGATTTCTACCGCAATATGCGTATTCAGGATCTGGCACAAGGGATCCATAAGCTGATTCGTAA
ACACGATCTTCCCGGTTTGATGTTGCGGGCATTCGATACTTTGCCGGAGATGATCATGACGCCACATC
AGGCATGGCAACGACAAATTAAAGGCGAAGTAGAAACCATTGCGCTGGAACAACTGGTCGGTAGAGTA
TCGGCAAATATGATCCTGCCTTATCCACCGGGCGTACCGCTGTTGATGCCTGGAGAAATGCTGACCAA
AGAGAGCCGCACAGTACTCGATTTTCTACTGATGCTTTGTTCCGTCGGGCAACATTACCCCGGTTTTG
AAACGGATATTCACGGCGCGAAACAGGACGAAGACGGCGTTTACCGCGTACGAGTCCTAAAAATGGCG
GGATAACTTGCCAGAGCGGCTTCCATTTACGCCCCGCCCTGTTGACAGCTTATCATCGATAAGCTTTA

ATGCGGTAGTTTATCACAGTTAAATTGCTAACGCAGTCAGGCACCGTGTATGAAATCTAACAATGCGC
TCATCGTCATCCTCGGCACCGTCACCCTGGATGCTGTAGGCATAGGCTTGGTTATGCCGGTACTGCCG
GGCCTCTTGCGGGATATCGTCCATTCCGACAGCATCGCCAGTCACTATGGCGTGCTGCTAGCGCTATA
TGCGTTGATGCAATTTCTATGCGCACCCGTTCTCGGAGCACTGTCCGACCGCTTTGGCCGCCGCCCAG
TCCTGCTCGCTTCCCTACTTGGAGCCACTATCGACTACGCGATCATGGCGACCACACCCGTCCTGTGG
ATCCTCTACGCCGGACGCATCGTGGCCGGCATCACCGGCGCCACAGGTGCGGTTGCTGGCGCCTATAT
CGCCGACATCACCGATGGGGAAGATCGGGCTCGCCACTTCGGGCTCATGAGCGCTTGTTTCGGCGTGG
GTATGGTGGCAGGCCCCGTGGCCGGGGGACTGTTGGGCGCCATCTCCTTGCATGCACCATTCCTTGCG
GCGGCGGTGCTCAACGGCCTCAACCTACTACTGGGCTGCTTCCTAATGCAGGAGTCCCATAAGGGAGA
GCGTCGACCGATGCCCTTGAGAGCCTTCAACCCAGTCAGCTCCTTCCGGTGGGCGCGGGGCATGACTA
TCGTCGCCGCACTTATGACTGTCTTCTTTATCATGCAACTCGTAGGACAGGTGCCGGCAGCGCTCTGG
GTCATTTTCGGCGAGGACCGCTTTCGCTGGAGCGCGACGATGATCGGCCTGTCCCTTGCGGTATTCGG
AATCTTGCACGCCCTCGCTCAAGCCTTCGTCACTGGTCCCGCCACCAAACGTTTCGGCGAGAAGCAGG
CCATTATCGCCGGCATGGCGGCCGACGCGCTGGGCTACGTCTTGCTGGCGTTCGCGACGCGAGGCTGG
ATGGCCTTCCCCATTATGATTCTTCTCGCTTCCGGCGGCATCGGGATGCCCGCGTTGCAGGCCATGCT
GTCCAGGCAGGTAGATGACGACCATCAGGGACAGCTTCAAGGATCCCTCGCGGCTCTTACCAGCCTAA
CTTCCATCATTGGACCGCTGATCGTCACGGCGATTTATGCCGCCTCCGCGAGCACATGGAACGGGTTG
GCATGGATTGTAGGCGCCGCCCTATACCTTGTCTGCCTCCCCGCGTTGCGTCGCGGTGCATGGAGCCG
GGCCACCTCCACCTGAAGGGCGGGGCGTAAATGGAAGCCGGCGGCACCTCGCTAACGGATTCACCACT
CCAAGAATTGGAGCCAATCAATTCTTGCGGAGAACTGTGAATGCGCAAACCAACCCTTGGCAGAACAT
ATCCATCGCGTCCGCCATCTCCAGCAGCCGCACGCGGCGCATCTCGGGCTCCTTGCAT

Protein sequence 1. strepTagII-SUMO-(tev)-dPTE2-H55
MGSSWSHPQFEKGSSMASMSDSEVNQEAKPEVKPEVKPETHINLKVSDGSSEIFFKIKKTTPLRRLME
AFAKRQGKEMDSLRFLYDGIRIQADQTPEDLDMEDNDIIEAHREQIGGIEENLYFQSNIGSGITNSGD
RINTVRGPITISEAGFTLMHEHICGSSAGFLRAWPEFFGSRDALAEKAVRGLRRARAAGVRTIVDVST
FDIGRDVELLAEVSEAADVHIVAATGLWFDPPLSMRLRSVEELTQFFLREIQYGIEDTGIRAGIIKVA
TTGKATPFQERVLRAAARASLATGVPVTTHTDASQRDGEQQADIFESEGLDPSRVCIGHSDDTDDLDY
LTALAARGYLIGLDHIPHSAIGLEDNASAAALLGLRSWQTRALLIKALIDQGYADQILVSNDWLFGFS
SYVTNIMDVMDRVNPDGMAFIPLRVIPFLREKGVPDETLETIMVDNPARFLSPTLRAS

Protein sequence 2. strepTagII-SUMO-(tev)-dPTE2-H55TAG
MGSSWSHPQFEKGSSMASMSDSEVNQEAKPEVKPEVKPETHINLKVSDGSSEIFFKIKKTTPLRRLME
AFAKRQGKEMDSLRFLYDGIRIQADQTPEDLDMEDNDIIEAHREQIGGIEENLYFQSNIGSGITNSGD
RINTVRGPITISEAGFTLM(ncAA)EHICGSSAGFLRAWPEFFGSRDALAEKAVRGLRRARAAGVRTI
VDVSTFDIGRDVELLAEVSEAADVHIVAATGLWFDPPLSMRLRSVEELTQFFLREIQYGIEDTGIRAG
IIKVATTGKATPFQERVLRAAARASLATGVPVTTHTDASQRDGEQQADIFESEGLDPSRVCIGHSDDT
DDLDYLTALAARGYLIGLDHIPHSAIGLEDNASAAALLGLRSWQTRALLIKALIDQGYADQILVSNDW
LFGFSSYVTNIMDVMDRVNPDGMAFIPLRVIPFLREKGVPDETLETIMVDNPARFLSPTLRAS

VI. References

(1) Caldwell, S. R.; Newcomb, J. R.; Schlecht, K. A.; Raushel, F. M. Limits of Diffusion in the Hydrolysis of Substrates by the Phosphotriesterase from Pseudomonas Diminuta. *Biochemistry* **1991**, *30* (30), 7438–7444. https://doi.org/10.1021/bi00244a010.

(2) Bigley, A. N.; Xiang, D. F.; Narindoshvili, T.; Burgert, C. W.; Hengge, A. C.; Raushel, F. M. Transition State Analysis of the Reaction Catalyzed by the Phosphotriesterase from Sphingobium Sp. TCM1. *Biochemistry* **2019**, *58* (9), 1246–1259. https://doi.org/10.1021/acs.biochem.9b00041.
